# Diverse Role of *bla*_CTX-M_ and Porins in Mediating Ertapenem Resistance among Carbapenem-Resistant Enterobacterales

**DOI:** 10.3390/antibiotics13020185

**Published:** 2024-02-13

**Authors:** Cody A. Black, Raymond Benavides, Sarah M. Bandy, Steven D. Dallas, Gerard Gawrys, Wonhee So, Alvaro G. Moreira, Samantha Aguilar, Kevin Quidilla, Dan F. Smelter, Kelly R. Reveles, Christopher R. Frei, Jim M. Koeller, Grace C. Lee

**Affiliations:** 1 College of Pharmacy, The University of Texas at Austin, Austin, TX 78712, USA; blackc1@uthscsa.edu (C.A.B.);; 2 Joe R. and Teresa Lozano Long School of Medicine, The University of Texas Health Science Center at San Antonio, San Antonio, TX 78229, USA; 3 Department of Pathology and Laboratory Medicine, The University of Texas Health Science Center at San Antonio, San Antonio, TX 78229, USA; 4 University Health System, San Antonio, TX 78229, USA; 5 College of Pharmacy, Western University of Health Sciences, Pomona, CA 91766, USA; 6 Veterans Administration Research Center for AIDS and HIV-1 Infection and Center for Personalized Medicine, South Texas Veterans Health Care System, San Antonio, TX 78229, USA

**Keywords:** CRE, ESBL, extended-spectrum beta-lactamase, non-carbapenemase-producing, ertapenem-resistant, meropenem-susceptible, porin loss, outer-membrane protein, mobile genetic elements, molecular epidemiology

## Abstract

Among carbapenem-resistant Enterobacterales (CRE) are diverse mechanisms, including those that are resistant to meropenem but susceptible to ertapenem, adding further complexity to the clinical landscape. This study investigates the emergence of ertapenem-resistant, meropenem-susceptible (ErMs) *Escherichia coli* and *Klebsiella pneumoniae* CRE across five hospitals in San Antonio, Texas, USA, from 2012 to 2018. The majority of the CRE isolates were non-carbapenemase producers (NCP; 54%; 41/76); 56% of all NCP isolates had an ErMs phenotype. Among ErMs strains, *E. coli* comprised the majority (72%). ErMs strains carrying *bla*_CTX-M_ had, on average, 9-fold higher copies of *bla*_CTX-M_ than CP-ErMs strains as well as approximately 4-fold more copies than *bla*_CTX-M_-positive but ertapenem- and meropenem-susceptible (EsMs) strains (3.7 vs. 0.9, *p* < 0.001). Notably, carbapenem hydrolysis was observed to be mediated by strains harboring *bla*_CTX-M_ with and without a carbapenemase(s). ErMs also carried more mobile genetic elements, particularly IS*26* composite transposons, than EsMs (37 vs. 0.2, *p* < 0.0001). MGE- IS*Vsa5* was uniquely more abundant in ErMs than either EsMs or ErMr strains, with over 30 more average IS*Vsa5* counts than both phenotype groups (*p* < 0.0001). Immunoblot analysis demonstrated the absence of OmpC expression in NCP-ErMs *E. coli*, with 92% of strains lacking full contig coverage of *ompC*. Overall, our findings characterize both collaborative and independent efforts between *bla*_CTX-M_ and OmpC in ErMs strains, indicating the need to reappraise the term “non-carbapenemase (NCP)”, particularly for strains highly expressing *bla*_CTX-M_. To improve outcomes for CRE-infected patients, future efforts should focus on mechanisms underlying the emerging ErMs subphenotype of CRE strains to develop technologies for its rapid detection and provide targeted therapeutic strategies.

## 1. Introduction

Carbapenem resistance in the Enterobacterales family poses a growing and pervasive threat to human health worldwide [1]. Despite advances in treatment strategies, these organisms continue to adapt, rendering them resistant to last-line antibiotics via a complex interplay of anti-carbapenem mechanisms [2,3]. While the mechanisms driving carbapenem resistance vary from region to region, the most measured and recognized mechanism is carbapenemase production, including serine carbapenemases (e.g., *bla*_KPC_) as well as metallo-β-lactamases (MBLs), such as New Delhi metallo-β-lactamase (*bla*_NDM_) [4].

However, the implications of carbapenem resistance occurring in strains that lack a carbapenemase (NCP) have been less studied. NCP-related infections have exhibited similar infection-related mortality and healthcare utilization as CPE-related infections [5]. While carbapenemase-producing Enterobacterales (CPE) is the predominant global driver of CRE, NCPE predominance has been emerging in some regions, including South Texas, with rates as high as 61% [6,7]. While an increasing rate of clinical laboratories have the capability to detect strains that harbor carbapenemases using currently available molecular rapid diagnostic tests, there is no such test to rapidly detect NCPE strains. This presents a major challenge for the timely diagnosis of a CRE infection, leading to delayed targeted treatment, overprescribing of antimicrobials, transmission, and poor outcomes. Moreover, NCPE is attributable to its diverse underlying mechanisms, which most frequently are combinatorial and concerted and cannot be detected by the presence/absence of a specific gene. It is suspected that higher production of cephalosporinases including extended-spectrum β-lactamase (ESBL) enzymes, like *bla*_CTX-M_, Ambler class C (e.g., *bla*_AmpC_), and certain variants of *bla*_SHV_ contribute to carbapenem resistance among NCP-CRE (4,5). However, additional concerted anti-carbapenem resistance mechanisms with cephalosporinase production, such as loss or altered outer membrane protein (Omp) impacting intracellular carbapenem concentration and rate of hydrolysis (level of activity of cephalosporinases) have been implicated and requires further investigation [5].

Moreover, among NCPEs are diverse mechanisms, including those that are resistant to either meropenem or imipenem–cilastatin but susceptible to ertapenem, adding further complexity to the clinical landscape. The clinical relevance is underscored as the Infectious Diseases Society of America (IDSA) treatment guidelines for Gram-negative infections provide specific recommendations for CRE infections that are resistant to ertapenem (MICs ≥ 2 mcg/mL) but susceptible to meropenem (MICs ≤ 1 mcg/mL) (ertapenem-resistant, meropenem-susceptible; ErMs) [8]. Mutations for ertapenem resistance have been shown to provide the genetic background for non-carbapenemase meropenem resistance [9]. However, investigations into the molecular and clinical profiles underlying the ErMs phenotype have been limited. Previous studies have demonstrated that high levels of ESBL-associated transposon insertional mutagenesis occur in ertapenem-resistant *K. pneumoniae* and ST-131 *E. coli* clinical strains, contributing to the evolution of meropenem resistance [9,10]. Consequently, clinicians rely on susceptibility testing results, which can take 3–5 days, before optimizing antibiotics. Herein, we report on mechanisms underlying the phenotypic emergence of ErMs *E. coli* and *K. pneumoniae*, with a particular focus on NCPE.

## 2. Results

### 2.1. ErMs Predominantly Harbor bla_CTX-M_ among NCPE

As previously reported, 99 CRE isolates from unique patients were collected from five hospitals in South Texas, United States, between 2011 and 2019 [7]. Of these, *E. coli* and *K. pneumoniae* comprised the majority (77%; 76/99), consisting of 47 *K. pneumoniae* and 29 *E. coli*. Antimicrobial susceptibility results for *E. coli* and *K. pneumoniae* isolates are shown in Table 1. Resistance to either ertapenem and/or meropenem was confirmed phenotypically. Overall, 38% (29) had an ErMs phenotype, while 62% (47) were ertapenem- and meropenem-resistant (ErMr). *E.coli* isolates had an ErMs phenotype more frequently than *K. pneumoniae* (72% vs. 17%; *p* < 0.001). Meropenem susceptibility was maintained by 44% of the CRE isolates. Piperacillin–tazobactam susceptibility was 19% and 35% overall and among ErMs CRE, respectively. Among other common antibiotics active against CRE, susceptibility rates were 77% (ceftazidime–avibactam), 98% (tigecyclin), 16% (levofloxacin), 23% (trimethoprim–sulfamethoxazole), 91% (amikacin), 95% (polymyxins), and 98% (imipenem–relebactam). Two *K. pneumoniae* (one NCP-ErMs and one CP-ErMS) and one NCP-*E. coli* were polymyxin B-resistant.

Short-read, whole genome sequence (WGS) analysis was used to annotate known resistance genes among all 76 *E. coli* and *K. pneumoniae* isolates (Table 2). Overall, 54% of CRE lacked a carbapenemase gene (NCPE), and 46% (35/76) were CPE. *E. coli* was more frequently NCPE than *K. pneumoniae* (76% vs. 40%; *p* = 0.01). Contrastingly, *K. pneumoniae* were more than twice as likely to harbor a carbapenemase gene than *E. coli* (Table 2), which predominantly comprised *bla*_KPC_ (23/28). *K. pneumoniae* also harbored a penicillinase *bla*_TEM_ and/or *bla*_SHV_ more frequently than *E. coli* (89% vs. 62%; *p* = 0.01). The ErMs vs. ErMr phenotype were more likely to be NCPE (83% vs. 36%, *p* < 0.001) and enriched for carrying *bla*_CTX-M_ (83% vs. 49%, respectively; *p* = 0.01). While CPE was more likely to be ErMr, 5 (14%) of CPE isolates were ErMs; four harboring *bla*_KPC_ and one *bla*_NDM_. Contrastingly, ErMr isolates were more commonly CPE than ErMs (64% vs. 17%, *p* < 0.001), with *bla*_KPC_ making up the majority of carbapenemase genes among this phenotype (51% vs. 14%, *p* = 0.002). In addition, CP strains carried *bla*_OXA-1_ or *bla*_OXA-9_ more frequently than NCPE strains (60% vs. 29%, *p* = 0.01).

The distribution of MBLs, oxacillinases, AmpC cephalosporinases, and ESBL genes was similar between *E. coli* and *K. pneumoniae*, with the exception that *bla*_SHV-12_ ESBL genes were solely carried by seven *K. pneumoniae* isolates. In total, 5 isolates harbored an MBL carbapenemase gene (2 *bla*_NDM-1_, 2 *bla*_NDM-5_, and 1 *bla*_VIM-27_), 28 harbored a *bla*_KPC_ gene (18 *bla*_KPC-2_ and 10 *bla*_KPC-3_), two harbored a *bla*_OXA-232_ carbapenemase gene, 33 harbored a narrow spectrum oxacillinase *bla*_OXA-1_ or *bla*_OXA-9_ gene (22 *bla*_OXA-1_ and 12 *bla*_OXA-9_), 52 harbored an ESBL, of which *bla*_CTX-M-15_ made up the majority (43 *bla*_CTX-M-15_, 3 *bla*_CTX-M-14_, 1 *bla*_CTX-M-27_, 7 *bla*_SHV-12_, and 1 *bla*_SHV-105_). *bla*_OXA-1_ or *bla*_OXA-9_ was co-harbored with *bla*_CTX-M-15_ in 27 (36%) of isolates (11 *E. coli* and 16 *K. pneumoniae*). Among *bla*_KPC_ harboring isolates, *bla*_OXA-1_ or *bla*_OXA-9_ was co-harbored in 14 (18%) of isolates (3 *E. coli* and 11 *K. pneumoniae*). Sixty (79%) of *E. coli* and *K. pneumoniae* carried a penicillinase gene (*bla*_TEM_ or *bla*_SHV_). Twelve (16%) *E. coli* and *K. pneumoniae* carried a class C cephalosporinase gene, with plasmid-mediated *bla*_CMY_ variants making up the majority (11/12).

### 2.2. ErMs E. coli Associates with Mobile Genetic Elements Interposed by bla_CTX-M_

Mobile genetic elements (MGEs), including insertion sequences (ISs), composite transposons, and other transposable elements, are associated with the mobilization of antibiotic resistance genes, including β-lactamases. We aimed to investigate the association between ISs and *bla*_CTX-M_ genes, particularly their genetic context among ErMs *E. coli*. To gain insight into MGEs total abundance and their associations with *bla*_CTX-M_ amplification and mobilization across three distinct carbapenem phenotypes, we annotated MGEs for five ErMs *E. coli* (EC-4, 6, 13, 30, and 35) and four ErMr *E. coli* (EC-5, 23, 67, and 68) using MobileElementFinder (https://cge.food.dtu.dk/services/MobileElementFinder/, accessed on 29 June 2023). For reference, five *bla*_CTX-M-_positive ertapenem- and meropenem-susceptible (EsMs) *E. coli* FASTA sequences (Accessions: GCA_032120475.1, GCA_032120375.1, GCA_032122895.1, GCA_032329675.1, GCA_031776215.1) were obtained from NCBI Isolates Browser (https://www.ncbi.nlm.nih.gov/pathogens/isolates, accessed on 29 June 2023). ErMs and ErMr were selected from our collection to match the various host sources of the EsMs (e.g., urine, blood, and sputum). To determine *bla*_CTX-M_ associated MGEs, our evaluation included MGEs that met two criteria on the same contig: (i) either interposed *bla*_CTX-M_ or (ii) were immediately upstream of *bla*_CTX-M_.

**Figure 1:**
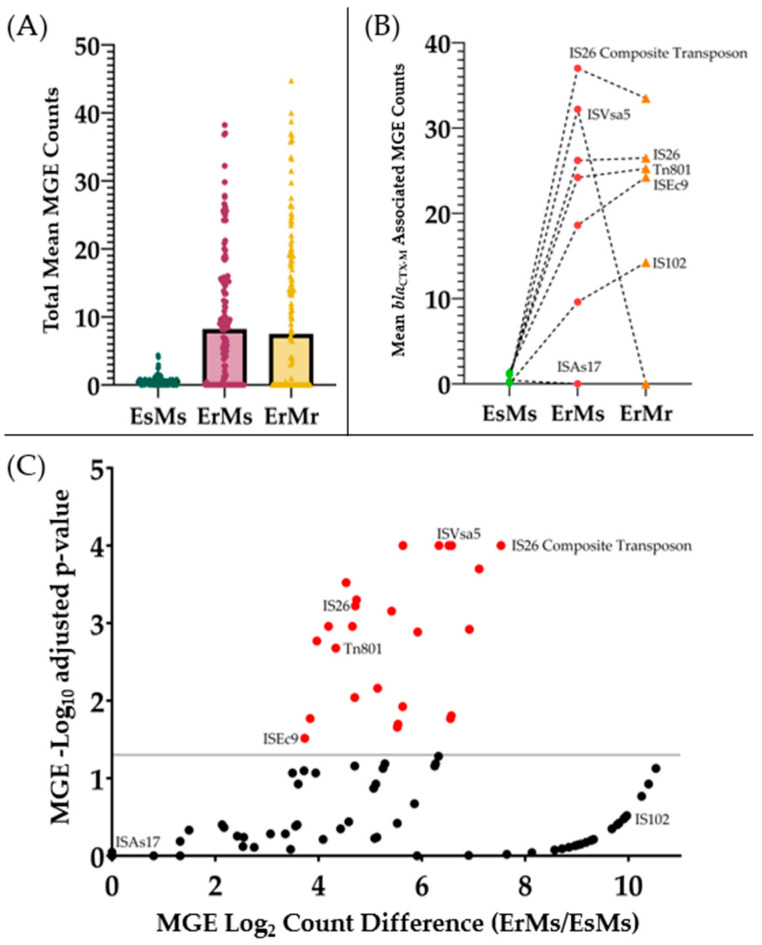
*bla*_CTX-M_-associated mobile genetic elements (MGEs) across three carbapenem phenotypes. Five ertapenem- and meropenem-susceptible (EsMs) with *bla*_CTX-M_, five ertapenem-resistant but meropenem-susceptible (ErMs), and four ertapenem- and meropenem-resistant (ErMr) *E. coli* were annotated for MGEs with MobileElementFinder database v1.0.2 (https://cge.food.dtu.dk/services/MobileElementFinder/, accessed on 29 June 2023). (**A**) Total annotation counts were compared across all phenotypes (Supplemental Data S1). (**B**) MGE annotations interposed by (composite transposons) or upstream from *bla*_CTX-M_ were counted and plotted across all phenotypes. (**C**) is a volcano plot comparing all MGE counts between EsMs and ErMs. Log2-fold count difference between ErMs and EsMs MGEs were plotted against Log10-transformed adjusted *p*-values (two-way ANOVA) of all MGEs between these two phenotypes. Values above 1.3 Log10 (*p* < 0.05; grey line) were considered statistically significant. All red MGEs are present at higher frequencies in ErMs than in EsMs *E. coli*.

ErMs *E. coli* had higher global mean MGE counts than EsMs (9.4 vs. 0.5, *p* < 0.001), but similar to ErMr strains (Figure 1A, Supplemental Data S1). A total of seven *bla*_CTX-M_ associated MGEs (i.e., MGE annotations interposed by composite transposons or upstream from *bla*_CTX-M_) were identified, including IS*26*, IS*26* composite transposon (IS*26* inverted repeat flanked unit), IS*Vsa5* (= IS*10R*), IS*Ec9*, Tn*801*, IS*102*, and IS*As17* (Figure 1B). When evaluating MGE Log_2_-transformed count differences between ErMs vs. EsMs (Figure 1C), five of these seven *bla*_CTX-M_-associated MGEs were significantly higher, including IS*26* composite transposon (mean count difference 36.8, *p* < 0.0001), IS*Vsa5* (31.8, *p* < 0.0001), IS*26* (25.2, *p* = 0.0006), Tn*801* (23, *p* = 0.002), and IS*Ec9* (17.2, *p* = 0.03). Across phenotype groups, a stepwise pattern of increasing *bla*_CTX-M_ associated MGE counts were observed from EsMs to ErMs to ErMr (Figure 1B). Notably, MGE- IS*Vsa5* was uniquely more abundant in ErMs than either EsMs or ErMr strains, with over 30 more average IS*Vsa5* counts than both phenotype groups (*p* < 0.0001) (Figure 1B,C). Comparing ErMs to ErMr showed a wide range of distinct MGEs more abundant in each phenotype (Supplemental Data S1 and S2, Figure S1).

### 2.3. Carbapenemase and bla_CTX-M_ Hastens Meropenem Hydrolysis in CPE and NCPE

To determine the effect of various β-lactamase profiles on carbapenem hydrolysis rates, intracellular meropenem concentrations were measured via parent molecule quantification over time using liquid chromatography–tandem mass spectrometry (LC-MS/MS). Nine representative isolates with diverse profiles were evaluated, including *bla*_NDM_ and *bla*_KPC_ harboring *E. coli* and *K. pneumoniae*, and *bla*_CTX-M-15_, *bla*_OXA-1_, *bla*_TEM_ harboring non-carbapenemase-producing *E. coli* isolates. Vaborbactam served as a secondary internal standard across all LC-MS/MS assays. The concentration of meropenem or vaborbactam (ng/mL) was compared at three time points (1, 2, and 18 h). Hydrolysis rates were determined using the formula, $\left(-\frac{∆\left[parent\right]}{∆t}\right)$, and reported as ng/mL-hour in Table 3. Of the nine isolates, three harbored *bla*_NDM_ (EC22, EC23, and KP26), three harbored *bla*_KPC_ (EC74, KP15, and KP56), and three were NCPE (EC68, EC5, and EC201).

Distinct rates of meropenem hydrolysis were observed. Isolates harboring *bla*_CTX-M_ displayed higher rates of meropenem hydrolysis across NCPE and CPE isolates (Table 3). Those harboring *bla*_NDM_ showed a rapid loss of meropenem; two isolates (EC22 and KP26) rapidly fell below the lower limit of quantitation (LLQ) within one hour, while the other isolate (EC23) displayed a rapid rate of meropenem hydrolysis over the over the 18 h experimental period (−2.8 ng/mL-hour). Among the *bla*_KPC_ harboring isolates (KP56, EC74, and KP15), meropenem hydrolysis was 1.7 times faster, on average, when *bla*_CTX-M_ was present (Table 3). Among the NCP isolates tested (EC5, EC68, and EC201), the two isolates that harbored *bla*_CTX-M-15_ displayed 1.8 times faster rates of meropenem hydrolysis than the non-*bla*_CTX-M-15_ isolate (EC68). Correspondingly, the rate of meropenem hydrolysis among the *bla*_CTX-M-15_ positive NCP isolates ranged between −0.5 to −1.0, including cases where an NCP (EC201) strain had rates similar to those of *bla*_KPC_-producing isolates KP56 (ATCC 1705) and EC74. Overall, meropenem hydrolysis was observed among CP and NCP isolates. Rates were highest among CP with the presence of the ESBL *bla*_CTX-M-15_.

Vaborbactam concentrations remained relatively constant over hours 1 to 18 with an average t_2_ − t_1_ concentration of +0.75 (±1.1) ng/mL. No vaborbactam hydrolysis was observed other than minor loss (−0.1 ng/mL) in EC68 (NCPE) over 18 h (Table 3, Supplement Data S2, Tables S1 and S2, and Supplement Data S3).

### 2.4. Ertapenem-Resistant E. coli and K. pneumoniae Carry Elevated Copies of bla_CTX-M_ Genes

The relative copy number (ΔCt) of *bla*_CTX-M_, *bla*_OXA-1/9_, *bla*_SHV_, *bla*_TEM_, *bla*_CMY_, and *bla*_KPC_ genes were quantified in a subset of eight *E. coli* and *K. pneumoniae* ErMs (EC12, EC30, EC31, EC35; KP10, KP38, KP45, and KP54) and eight ceftriaxone-resistant ESBL clinical strains which were ertapenem- and meropenem-susceptible (EsMs) (EC87, EC88, EC89, EC92; KP85, KP86, KP90, and KP91) (Table 4; Supplemental Data S2, Table S4) using quantitative polymerase chain reaction (qPCR). A species-specific primer for the *rpsL* gene was used as the control gene in both ErMs and EsMs strains. Fold copies were calculated with the formula ΔCt = 2^(CT*rpsL* − CTtarget)^ relative to *rpsL* of the same isolate. Overall, the largest copy number difference between the two phenotypes was in *bla*_CTX-M_, with a mean difference of 12-fold more log_2_-transformed copies in ErMs (17.1 vs. 4.8; see Table 4). The mean differences between all other targeted genes were within one log_2_-transformed fold. All *bla*_CTX-M_-positive ErMs *E. coli* (4/4) and *K. pneumoniae* (3/4) co-harbored *bla*_OXA-1_, *bla*_SHV_, and/or *bla*_TEM_. All ErMs harbored *bla*_TEM_, regardless of species. This is in contrast to EsMs, where the majority (5/8) were *bla*_TEM_ negative. *bla*_SHV_ was solely harbored by *K. pneumoniae*, regardless of phenotype. *bla*_CMY_ was detected in one ErMs and two EsMs. *bla*_KPC_ was detected in two ErMs, EC12, a clinical strain, and KP54, an ATCC strain with a distinct subpopulation of KPC producers (*Klebsiella pneumoniae* (Schroeter) Trevisan BAA-1903; https://www.atcc.org/api/pdf/product-sheet?id=BAA-1903, accessed on 29 June 2023).

**Figure 2:**
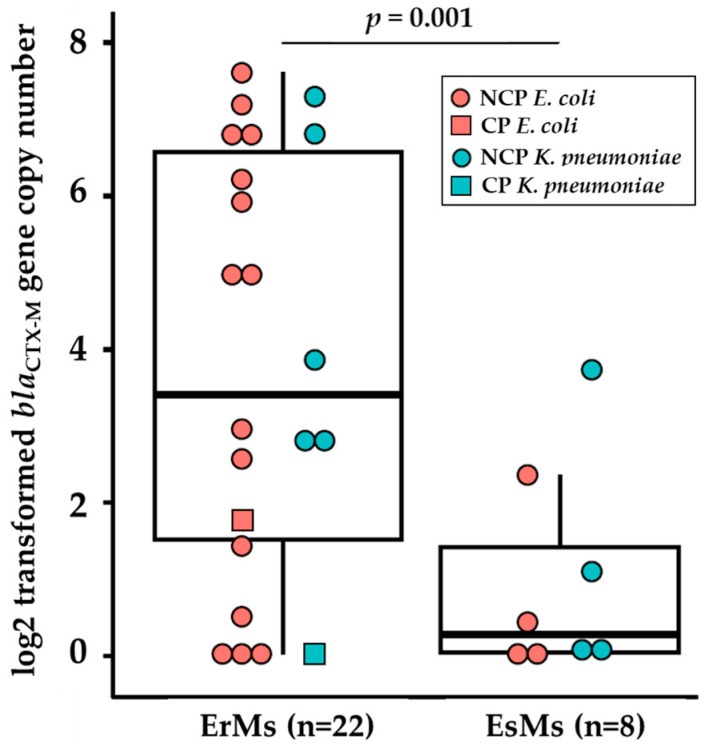
Mean log_2_-transformed *bla*_CTX-M_ gene copy number by ertapenem and meropenem phenotype. ΔΔCt =2^(ΔCTcontrol−ΔCTtarget)^ was used to calculate copy number, using *rpsL* gene as the control gene and EC87 (a ceftriaxone-resistant but ertapenem- and meropenem-susceptible (EsMs) strain) as the control strain (*bla*_CTX-M_ ΔCt of 1.0; log2 ΔΔCt = 0.0). Abbreviations: *bla*: β-lactamase; EsMs: ertapenem- and meropenem-susceptible but ceftriaxone-resistant; ErMs: ertapenem-resistant, meropenem-susceptible. Performed t-test for fold change difference between ErMs and EsMs.

Based on these data, we quantified the log_2_-transformed ΔΔCt of *bla*_CTX-M_ among a larger set of ErMs, using the formula ΔΔCt = 2^(ΔCTcontrol − ΔCTtarget)^. The EsMs *E. coli* isolate EC87 was used as the *bla*_CTX-M_ control strain as it harbored a single copy of *bla*_CTX-M_ relative to *rpsL* with a log_2_ ΔΔCt of zero. We examined sixteen ErMs *E. coli* (Figure 2), six ErMs *K. pneumoniae*, four EsMs *E. coli,* and four EsMs *K. pneumoniae*. Overall, 82% (18) of the 22 ErMs harbored *bla*_CTX-M-15_ or *bla*_CTX-M-14_, while the four remaining ErMs had no detectable *bla*_CTX-M_ (Figure 2). Furthermore, ErMs isolates harboring *bla*_CTX-M_ carried 4-fold more log_2_-transformed copies of *bla*_CTX-M_ than ceftriaxone-resistant EsMs (3.7 vs. 0.9, *p* < 0.001) across both species and carbapenemase status. Interestingly, NCP-ErMs had 3-fold more *bla*_CTX-M_ copies than CP-ErMs (4.0 vs. 0.8) (Figure 2).

### 2.5. Porin Alterations Are Frequent among Ertapenem-Resistant NCPE E. coli

Porin and efflux genes of *E. coli* (*ompC*, *ompF*, and *tolC*) and *K. pneumoniae* (*ompK35*, *ompK36*, and *oqxA*) were identified and quantified using qPCR relative to *rpsL* across the same eight ErMs and eight EsMs (Table 4). Porin genes were detected in all strains except two *K. pneumoniae* EsMs, which had no detectable *ompK35* (KP86 and KP91). Across all tested strains, there were 0.7-fold more log_2_-transformed fold copies of porin genes relative to *rpsL*, ranging from 0.0 to 1.8-fold. Comparing ΔCt of all porins regardless of species, ErMs had more log_2_-transformed fold copies than EsMs (0.89× vs. 0.51×; *p* = 0.001). No porin copy number difference was identified when stratified by species alone. The chromosomal efflux gene of *E. coli* (*tolC*) and the plasmid efflux gene of *K. pneumoniae* (*oqxA*) were also examined with qPCR. All isolates had detectable efflux genes except KP85. The mean log_2_-transformed copies of efflux genes were 0.97, ranging from undetectable to 1.9-fold higher than *rpsL*. The overall strength of the relationship between ΔCt of *ompC* and *bla*_CTX-M_ among all eight isolates was moderately positive (R^2^ = 0.4), with ertapenem-resistant strains showing less correlation (EsMs vs. ErMs; 0.8 vs. 0.3), particularly at *bla*_CTX-M_ ΔCt > 10 (Table 4; Supplemental Data S2, Figure S5).

The above analysis indicates that minimal differences in porin gene copy numbers were observed between ErMs vs. EsMs; we next evaluated sequence mutations outside of the qPCR primer sequence that may be present at different rates. In order to examine this, we aligned short-read sequences to a reference genome, *E. coli* str. K-12 substr. MG1655 (GenBank Accession: U00096) and *K. pneumoniae* CP000647. Porin gene alterations were then translated and categorized into three major amino acid variant categories, including (1) insertions and/or deletions, (2) frameshifts, or (3) premature stops.

Amino acid variants in *ompF*-like (*ompF*/*ompK35*) and *ompC*-like (*ompC*/*ompK36*) porin genes in CP-ErMr and NCP-ErMs *E. coli* and *K. pneumoniae* isolates are summarized in Table 5. Results were stratified by species as distinct porin alteration rates occur between *E. coli* vs. *K. pneumoniae*. All eight (100%) of ErMs *K. pneumoniae* were NCP, while 76% (16/21) of the *E. coli* ErMs were NCP, and 24% (5/21) of *E. coli* ErMs harbored *bla*_KPC-2_, *bla*_KPC-3_, or *bla*_NDM-5_.

Overall, porin variants were not detected in any of the CP-ErMr *E. coli* and in only 3.6% of the CP-ErMr *K. pneumoniae*. A translated amino acid alteration from either *ompC* or *ompF* sequences was significantly more frequent in NCP-ErMs *E. coli* than CP-ErMr *E. coli* (*p* = 0.002). Contrastingly, translated porin gene alterations were both more frequent and similar in alteration type (insertion/deletion, frameshift, and premature stop) in NCP-ErMs vs. CP-ErMr *K. pneumoniae* isolates, regardless of porin gene type (*ompK35* or *ompK36*).

In *K. pneumoniae*, premature stop codons in *ompK35* or *ompK36* genes occurred in 89% and 100% of CP-ErMr and NCP-ErMs isolates, respectively, with similar rates in individual porin genes. The most frequent premature stop codon positions in *ompK35* porin genes were p213* and p89*, occurring in 30% and 26%, respectively. In *ompK36* genes, p271* was the most frequent position of a premature stop codon. Concurrent *ompK35* and *ompK36* premature stop codons occurred in 57% (27/47) of all *K. pneumoniae* isolates. In addition, insertion/deletion (indel) and frameshift alterations occurred at similar rates in *ompK36* genes, regardless of carbapenemase status and phenotype. This is in contrast to *ompK35*, which was free of any indels or frameshifts among CP-ErMr and NCP-ErMs *K. pneumoniae* (Table 5).

All NCP-ErMs *E. coli* contained frameshift alterations, whereas these were not observed in either of the two CP-ErMr *E. coli* isolates (100% vs. 0%; *p* = 0.002; Table 5). Frameshifts were detected in *ompC* or *ompF* in 88% and 50% of NCP-ErMs *E. coli*, respectively. Similarly, *ompC* or *ompF* indels occurred in 63% of NCP-ErMs *E. coli* and none of the CP-ErMr *E. coli*. A premature stop codon was detected in one *E. coli*, which occurred in the *ompC* gene of an NCP-ErMs isolate.

In addition to these major translated porin gene alterations (indel, frameshift, and premature stop), translated missense amino acid changes were mapped to the protein databank (PDB) coordinate files of OmpF, OmpC, OmpK35, and OmpK36 (PDB: 4GCS, 7JZ3, 5o77, and 6RD3). The non-synonymous residue alterations predominantly related to external facing vestibular loops, including Loop 3, within OmpC/OmpK36 and OmpF/OmpK35 (Supplemental Data S2, Figure S2). In addition, frameshift mutations occurred most frequently within the Loop 4-β8-Loop 5 extracellular facing vestibule region, primarily in NCPE isolates. Of note, a GG, PT, or the previously reported GGD insertion within the conserved Loop 3 region (amino acid positions 133–136) of OmpK36 occurred solely among the *K. pneumoniae* clones 258 and 307, while *E. coli* Loop 3 nucleotides contained various missense changes only.

Overall, the frequency and type of translated porin alterations among ErMr and ErMs *K. pneumoniae* were not different. In contrast, ertapenem resistance seems to be related to *ompC* alterations among NCP-ErMs *E. coli.* Next, the coverage of the *ompC* gene was assessed in 26 *E. coli* (20 NCP, 6 CP) by viewing the mapped reads coverage and annotating low coverage areas, defined as areas where coverage falls below two standard deviations from the mean coverage (Table 6, Supplemental Data S2, Figure S4, and Supplemental Data S4). Of the *E. coli* genomes visualized, 62% (16/26) had a no-to-low read coverage region within the *ompC* gene averaging 103 ± 61 bp long, ranging from 7 bp to 173 bp in length across all visualized genomes. MG1655 K12 *E. coli* was used as mapping reference; accession: U00096 (Nucleotide [Internet]. Bethesda (MD): National Library of Medicine (US), National Center for Biotechnology Information; [1988]—[accessed on 11 November 2023]. Available from: https://www.ncbi.nlm.nih.gov/nuccore/U00096.2, accessed on 11 November 2023).

**Table 6:** Summary of ErMs *E. coli bla*_CTX-M_ copy number, *ompC* contig coverage, and OmpC status.

ID	Carbapenemase Status	*bla*_CTX-M_ Δ∆Ct ^A^	Contig Coverage ^B^ at K12 *ompC*	OmpC Band ^C^
EC12	CP (*bla*_KPC_)	+1.7	No gap (Full coverage)	Detected
EC14	CP (*bla*_KPC_)	+0.5	No gap (Low at c.539–c.545)	Detected
EC13	CP (*bla*_KPC_)	+5.0	No gap (Full coverage)	ND
EC75	CP (*bla*_KPC_)	ND	No gap (Full coverage)	Detected
EC30	NCP	+6.2	149 bp gap (c.424—c.531)	ND
EC31	NCP	+2.9	29 bp gap (c.544–c.531)	ND
EC35	NCP	+5.9	144 bp gap (c.429–c.531)	ND
EC2	NCP	+4.9	149 bp gap (c.424–c.531)	ND
EC3	NCP	+6.6	173 bp gap (c.416–c.515)	ND
EC32	NCP	+1.4	150 bp gap (c.424–c.530)	ND
EC33	NCP	+7.5	149 bp gap (c.424–c.530)	ND
EC34	NCP	ND	139 bp gap ^D^ (c.434–c.531)	ND
EC36	NCP	+7.1	140 bp gap (c.434–c.530)	ND
EC4	NCP	+6.8	No gap (Full coverage)	ND
EC6	NCP	+2.5	141 bp gap (c.431–c.532)	ND
EC66	NCP	ND	149 bp gap (c.424–c.531)	ND

**Figure 3:**
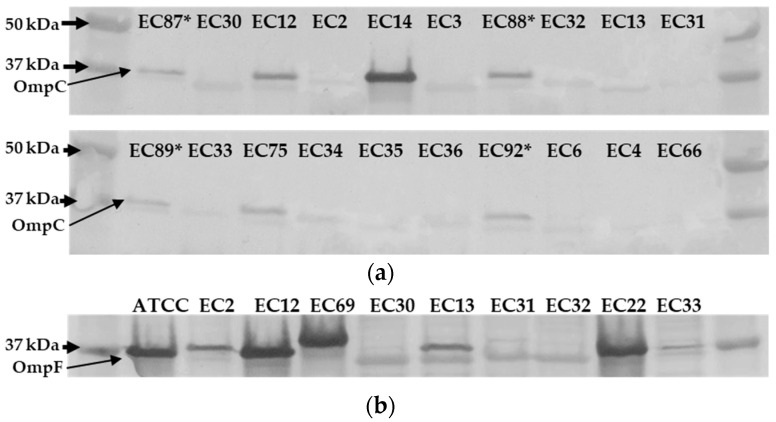
Immunodetection of OmpC and OmpF in ertapenem-resistant *E. coli* clinical strains. Total proteins were resolved by 4–15% SDS-PAGE. The proteins were electro-transferred to nitrocellulose membrane and immunodetected with polyclonal antibodies directed against denatured OmpC and/or OmpF porins. Only the relevant part of the blot is shown. (**a**) Immunodetection of OmpC in ertapenem-resistant, meropenem-susceptible (ErMs) *E. coli* clinical strains (*n* = 16). Isolates EC87*, EC88*, EC89*, and EC92* are ertapenem-susceptible, ceftriaxone-resistant (EsMs) *E. coli* clinical isolates. ATCC 2340 was used as positive control. Thick black arrows indicate molecular weights, and thin black arrows indicate the region of OmpC. (**b**) Immunodetection of OmpF and OmpC in ErMs *E. coli* clinical strains (*n* = 9). Thick black arrows indicate molecular weight, and thin black arrows indicate the region of OmpF.

Summary of ErMs *E. coli bla*_CTX-M_ copy number variations, *ompC* mapped reads coverage, and OmpC expression status. ^A^ Δ∆Ct = 2^(CTcontrol−CTtarget)^ was used to calculate copy number, using rpsL gene as the control gene and EC87 (a ceftriaxone-resistant but ertapenem- and meropenem-susceptible (EsMs) strain) as the control strain. ^B^ Contigs were de novo assembled, dissolved, and mapped to K12 (accession: U00096). If multiple gaps were detected, the largest was reported. ^C^ Total protein was prepared as a lysate, normalized, electrophoretically separated on a 4–15% gel, and detected with anti-OmpC rabbit antibodies (Figure 3a). ^D^ In addition to the noted *ompC* gap, EC34 had a 5570 bp gap spanning from c.343 of *ompC* to adjacent genes downstream. Abbreviations: BP: base pair; CP: carbapenemase-producing; NCP: non-carbapenemase-producing; ND: not detected.

*ompC* lesions were highly similar among all strains, spanning from c.416 to c.554, with c.531 occurring at the terminal end of the gap in 50% of sequences. NCP-*E. coli* represented 77% (20/26) of the visualized sequences and made up 94% (15/16) of the sequences with *ompC* coverage gaps (Table 6). ErMs and ErMr made up 81% (13/16) and 19% (3/16) of these *ompC* lesioned strains, respectively. Despite this, the frequency of *ompC* alignment gaps among ErMs (13/21) vs. ErMr (3/5) *E. coli* was not significantly different. Of the 10 strains that had complete *ompC* coverage (no hits on the low coverage annotation track; EC-4, 12, 13, 14, 22, 23, 29, 67, 69, and 75), the majority were CP (60%) comprising one CP-ErMr (EC23) and five CP-ErMs (EC-12, 13, 14, 22, and 75). No *ompC* lesions were noted in four NCP *E. coli* (EC-4, 29, 67, and 69). Overall, this highlights a distinct ompC genomic structure among CP vs. NCP *E. coli*. The lack of *ompC* lesions among visualized CP-*E. coli*, regardless of *bla*_KPC_ or *bla*_NDM_, is contrasted with their occurrence in the majority of NCP-*E. coli* isolates (0% vs. 80%, respectively; *p* < 0.001), indicating an important role of *ompC* genetic disruption among NCP *E. coli*.

### 2.6. Ertapenem-Resistant E. coli Lack OmpC Outer Membrane Protein

Although *ompC* genetic lesions seem to be related to *E. coli’s* non-carbapenemase-producing status rather than carbapenem phenotype (i.e., ErMs vs. ErMr), the level of OmpC protein expression among ErMs is unknown. To examine OmpC outer membrane protein abundance among ErMs, we used sodium dodecyl sulfate–polyacrylamide gel electrophoresis (SDS-PAGE) and immunodetection with anti-OmpC and anti-OmpF primary antibodies (ThermoFisher). Major porin OmpC presence/absence was evaluated in a subset of 16 representative ErMs *E. coli* isolates with four EsMs *E. coli* as OmpC control strains (EC87, 88, 89, and 92). These EsMs clinical strains were used as controls as they carried qPCR-confirmed *bla*_CTX-M_ yet remained ertapenem-susceptible. Overall, 4 of the ErMs isolates were CP-ErMs *E. coli* (3 *bla*_KPC-2_ and 1 *bla*_KPC-3_), while 12 were NCP-ErMs *E. coli*. See Table 6 for a summary of genomic and immunodetection results.

All four control EsMs had detectable OmpC bands (Figure 3a). OmpC was not detected in 81% (13/16) of tested ErMs *E. coli*. The three lanes in which OmpC was detected were loaded with EC12, EC14, and EC75, which are all *bla*_KPC_-producing ErMs *E. coli*. In fact, 75% (3/4) of the electrophoretically separated CP-ErMs *E. coli* lysates had a detectable OmpC band. Furthermore, the one CP-ErMs that did not have a detectable OmpC band (EC13) had 5-fold more genetic copies of *bla*_CTX-M-15_ than the EsMs control (Table 6). NCP-ErMs *E. coli* made up 75% (12/16) of the samples tested for OmpC separation (EC-2, 3, 4, 6, 30, 31, 32, 33, 34, 35, 36, and 66). No OmpC band was detected in any of these samples.

A combination of anti-OmpF and anti-OmpC primary antibodies (multiplexed) were used on representative ErMs *E. coli* isolates and ATCC 2340 (Figure 3b). It is evident that a band below OmpC (40 kDa) and around 37 kDa was visible in 6/9 of the isolates (EC2, EC13, EC30, EC31, EC32, and EC33). However, the other three isolates (ATCC 2340, EC12, EC22, and EC69) had very strong signals despite protein concentration normalization, making the OmpC/F distinction difficult to interpret.

## 3. Discussion

To thwart the potential mistreatment of patients inflicted with NCPE and/or ErMs CRE infections, more insight into the anti-carbapenem resistance mechanisms employed by these nefarious pathogens is urgently needed. This is especially true for infectious diseases caused by ErMs—a phenotype with significant clinical implications.

This study revealed that ErMs *E. coli* genomes contain more total MGEs counts than EsMs (Figure 1A), particularly *bla*_CTX-M_ associated MGEs, including IS*26*, IS*26* composite transposon, IS*Vsa5* (=IS*10R*), IS*Ec9*, and Tn*801* (Figure 1B,C). These MGEs were found to be either interposed by or directly adjacent to *bla*_CTX-M_ in ErMs *E. coli*. IS*26* is recognized for its frequent mobilization of antimicrobial resistance genes as “translocatable units,” inserting them adjacent to other IS*26* copies in Gram-negative bacteria. The *bla*_CTX-M_ genes are often associated with IS*26*-interrupted transposable elements positioned upstream from IS*Vsa5*, synonymous with IS*10R*, the active element in the plasmid-associated transposon Tn*10* [14]. Moreover, IS*10R* has demonstrated internal promoter regions in previous work [14]. As IS*Vsa5* was uniquely significantly more abundant among ErMs than either EsMs or ErMr strains, this insertion sequence may be playing a significant role in regulating *bla*_CTX-M_ expression in ErMs *E. coli*. Additionally, qualitative results of *bla*_CTX-M_ (presence/absence) in patient samples may be insufficient—rather, it is necessary to quantify the number of copies harbored by *E. coli* and *K. pneumoniae* as elevated copies can relate to ertapenem resistance (Figure 2). This is in contrast to carbapenemases, where determining the presence/absence of the gene seems to be sufficient to relate to ertapenem resistance, as single copies were able to produce ertapenem and meropenem resistance efficiently.

**Figure 4:**
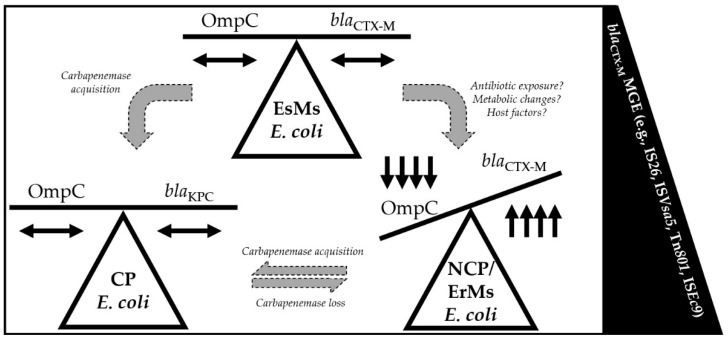
Diagram depicting the balance between β-lactamase copy number, OmpC expression (present/absent), and mobile genetic element abundance among carbapenemase-producing (CP), non-carbapenemase-producing (NCP), ertapenem-resistant, meropenem-susceptible (ErMs), and ceftriaxone-resistant but ertapenem- and meropenem-susceptible (EsMs) *E. coli*. Black double-headed arrows represent wildtype expression or copy number of OmpC protein or β-lactamase gene; black single-headed arrows indicate up or down expression or copy number compared to wildtype; large grey arrows represent theoretical changes required among EsMs *E. coli* to develop an ErMs phenotype; small grey single pointed arrows represent theoretical changes required among ErMs *E. coli* to develop a CP or NCP genotype. As carbapenemase enzymes, like *bla*_KPC_, are harbored in *E. coli*, OmpC expression is maintained in the presence of carbapenem exposure. However, if *bla*_CTX-M_ is present in NCP *E. coli*, carbapenem exposure drives an increase in *bla*_CTX-M_ copies and a decrease in OmpC expression.

In conjunction with *bla*_CTX-M_ expression, OmpC loss is evidently critical for the development of the ErMs phenotype among *E. coli*, as 75% of ErMr and 100% of EsMs but none of the ErMs isolates maintained OmpC (Figure 3a). Additionally, all *bla*_KPC_ producers with a single *bla*_CTX-M_ copy had detectable OmpC bands, but when multiple *bla*_CTX-M_ copies were detected (5-fold more than EsMs), no OmpC was detected in one isolate (EC13) (Figure 3a, Table 6). Taken together, this provides evidence that a collaborative effort between *bla*_CTX-M_ and OmpC occurs to result in ertapenem but not meropenem resistance and NCP *E. coli* (Figure 4).

As *bla*_CTX-M_ seems to be a less efficient carbapenem hydrolyzing enzyme than *bla*_KPC_ and *bla*_NDM_ (Table 4), insertion sequence disruption or other events may be triggered to disrupt OmpC expression. As *ompC* genomic lesions were more associated with NCP status than with ertapenem phenotype (i.e., ErMs vs. ErMr), it is more likely that the ErMs phenotype is an outcome of transcriptional or translational regulation. Another related potential cause of OmpC loss among the ErMs *E. coli* immunoblotted (Figure 3) is that 50% (8/16) of the isolates came from urinary sources (Supplement Data S2, Table S3), and seven out of those eight (88%) had no detectable OmpC. The previous literature has demonstrated that high osmolarity can cause the transcriptional downregulation of outer membrane proteins via the envZ/OmpR system [15,16]. However, in these environments, OmpF tends to be more labile than OmpC. Loss of OmpF seems to be less critical for the development of ErMs *E. coli* as the majority maintained a visible OmpF band (Figure 3b).

Within this CRE collection, *E. coli* was NCPE and/or had an ErMs phenotype more frequently than *K. pneumoniae* (Table 2). The fact that 72% (21/29) of the collected *E. coli* displayed an ErMs phenotype has important implications for current practice, as ertapenem susceptibility is selectively “suppressed” or not reported on microbiological reports coinciding with antimicrobial stewardship efforts to reduce ertapenem use in some hospitals [17]. Not reporting the ertapenem phenotype may lead to mistreatment of patients infected with this CRE subtype. As ESBL-producing Enterobacterales (ESBL-E) are on the rise in our area and globally, it may be prudent to test and report ertapenem results along with reducing the use of cephalosporins, like ceftriaxone, to possibly foil the rise of *bla*_CTX-M_ copy number variant ErMs strains.

*K. pneumoniae* was more commonly carbapenemase-producing, with *bla*_KPC_ being the most prevalent carbapenemase among the species (Table 2). In addition, *bla*_SHV_ and *bla*_TEM_ genes were amidst *K. pneumoniae* genomes more frequently than *E. coli* genomes. *bla*_OXA-1/9_ genes were also more commonly associated with CPE than NCPE. These co-harbored β-lactamase genes have been reported previously and seem to mobilize on modular genomic elements regularly [18,19]. In addition, *bla*_OXA-1/9_ has been previously associated with piperacillin–tazobactam resistance [10,19], which was reflected in this collection. Specifically, 10/12 (83%) of the *bla*_OXA-1/9_ positive ErMs were piperacillin–tazobactam-resistant. All *bla*_OXA-1/9_ positive ErMs co-harbored *bla*_CTX-M-15_.

These data also provide insight into the enzymatic efficiency of β-lactamases across the Ambler classes. A pattern of increased hydrolysis was measured in pathogens harboring *bla*_CTX-M_, *bla*_KPC_, and *bla*_NDM_. Excluding isolates that co-harbored any two of these three enzymes, an average meropenem hydrolysis rate was (−0.9 ng/mL-hour) for *bla*_CTX-M_ positive isolates and (−1.2 ng/mL-hour) for *bla*_KPC_ positive isolates; a 1.3 times increase in hydrolysis in *bla*_KPC_ vs. *bla*_CTX-M_ carrying isolates. All *bla*_NDM_ positive isolates co-harbored *bla*_CTX-M_, with two of these isolates achieving loss of meropenem below the lower limit of quantitation (LLQ) within the first hour. Examining the three NCPE-tested isolates, a 1.8 times increase of meropenem hydrolysis was measured in *bla*_CTX-M_ (EC5 and EC201) vs. non-*bla*_CTX-M_ (EC68) carrying isolate(s). These data suggest that the canonical attribution of “non-carbapenemase” to be reconsidered for *bla*_CTX-M_ positive isolates, as *bla*_CTX-M_ copy number variant strains can result in carbapenem hydrolysis and resistance.

In order to apply these findings to future work, it is important to consider the limitations associated with this study. Although we hypothesize that OmpC loss seems to be driven by genetic lesions which result in coverage gaps within the mid-range of the gene, this phenomenon can occur from many biological or environmental mechanisms, including mobile genetic element mediated disruption of *ompC* [10] and osmolarity. In addition, depending on the reference genome used to map contigs against, different coverage scores could be seen. When reviewing the multiplexed immunoblot (Figure 3b), the primary antibodies used may have some cross-reactivity due to the similarity of epitopes; however, when used alone (Figure 3a), the molecular weights aid in qualitative analysis of the bands. In terms of LC-MS/MS assays, β-lactams are very labile chemicals as they are prone to hydrolyzation unless stringent protocols are followed. Because of this, meropenem hydrolysis results may have been susceptible to non-β-lactamase degradation. It was attempted to control for by nulling out baseline hydrolysis of the parent molecule. Also, the contribution of a single β-lactamase is difficult in clinical isolates, which harbor multiple classes of β-lactamases without working with isogenic strains. Also, since the β-lactamase copy number was not calculated in these nine isolates, it is unclear if increased copies of these genes affected the meropenem hydrolysis rates, although most studies do not determine copy number variation in β-lactamase genes. In terms of clinically relevant limitations, the fact that a large portion of the collected CRE was from urinary sources makes extrapolation to non-urinary infections difficult. Moreover, there may be host and epidemiological features that predispose certain individuals [20,21,22] as well as unique vs. shared factors associated with specific sub-phenotypes of CRE that were beyond the scope of assessment [7,23,24]. Finally, we applied a wide array of techniques, including short-read, whole genomic data in conjunction with LC-MS/MS, qPCR, and Western blotting techniques to provide molecular characterization of ErMs *E. coli* and *K. pneumoniae*. However, there is a pressing need for the development of rapid and efficient diagnostic platforms that can be seamlessly integrated into clinical practice to enhance outcomes associated with these resistant pathogens [25,26].

In conclusion, the ErMs phenotype seems to be related to elevated gene copies of *bla*_CTX-M-14_ and *bla*_CTX-M-15_, especially when concurrently present with *ompC* genetic lesions and loss of OmpC production. Future efforts to characterize the molecular mechanisms that promote OmpC loss and quantification of *bla*_CTX-M_ among CRE will potentially improve patient care and mitigate further expansion of ertapenem resistance among patients afflicted with *E. coli* and/or *K. pneumoniae* infections.

## 4. Materials and Methods

### 4.1. Bacterial Isolates and Antimicrobial Susceptibility Testing

Carbapenem-resistant *E. coli* (*n* = 29) and *K. pneumoniae* (*n* = 47) isolates were examined from a previously collected biorepository of 99 CRE from 85 unique patients admitted to five different hospitals in South Texas, USA, between 2011 and 2019. Clinical isolates were stored at the time of carbapenem resistance discovery following Clinical and Laboratory Standards Institute (CLSI) standards and clinical laboratory procedures (e.g., positive Modified Hodge test, rapid antimicrobial resistance gene detection) (CLSI M100-ED33:2023 Performance Standards for Antimicrobial Susceptibility Testing, 33rd Edition). All isolates were initially speciated via biochemical assays and/or mass spectrometry at the clinical laboratory. Repeat confirmatory speciation was determined via WGS-KMER analysis. Abiding by current CDC definitions, CRE in this study was defined as Enterobacterales isolates resistant to any carbapenem or determined to be carbapenemase positive. The in vivo sources of the isolates varied (Supplement Data S2, Table S3). MICs of the isolates at the time of patient hospitalization were abstracted from electronic medical records and confirmed with microdilution susceptibility testing using the Sensititre™ Gram-Negative GNX2F AST Plate. Discrepancies in phenotypes were present among a small number of isolates (~2%), which were primarily *K. pneumoniae* and were interpreted as ErMs in the medical chart but ErMr upon repeat testing. These isolates were annotated as ErMr in downstream analysis. Carbapenem non-susceptibility was defined based on CLSI breakpoints: ertapenem- and meropenem-susceptible but ceftriaxone-resistant (EsMs): ertapenem ≤ 0.5 mcg/mL, meropenem MIC ≤ 1 mcg/mL and ceftriaxone MIC ≥ 4 mcg/mL; ErMs: ertapenem ≥ 1 mcg/mL and meropenem MIC ≤ 1 mcg/mL (ertapenem intermediate breakpoint annotated as resistant); ertapenem- and meropenem-resistant (ErMr): ertapenem ≥ 2 mcg/mL and meropenem MIC ≥ 4 mcg/mL (CLSI M100-ED33:2023 Performance Standards for Antimicrobial Susceptibility Testing, 33rd Edition). CRE with carbapenemase genes detected were termed CPE; those without carbapenemase genes were termed NCPE. *E. coli* strains ATCC 25922, and BAA-2340 were used as carbapenem-susceptible and carbapenem-resistant (*bla*_KPC-_producing) controls, respectively. *K. pneumoniae* strains BAA-1705, BAA-1706, and BAA-1903 were used as *bla*_KPC_-producing, non-carbapenemase-producing and ErMs controls, respectively.

### 4.2. Whole Genome Sequencing

For WGS, total bacterial DNA was extracted using a DNeasy PowerSoil kit (Qiagen, Redwood City, CA, USA). For qPCR, genomic and plasmidic DNA were extracted by following the CDC boil BacDNA Lysate protocol. WGS was conducted on all isolates using a NextSeq 500 sequencing instrument (Illumina Inc., San Diego, CA, USA) with 150-base paired-end reads (UT Health San Antonio, San Antonio, TX, USA), as previously described [7,27,28]. All short-read data and metadata were deposited in the NCBI BioProject (PRJNA1049776). Briefly, de novo assembly, variant analyses, and contig coverage visualization were conducted using CLC Genomics Workbench 20.1 (Qiagen, Redwood City, CA, USA) and Geneious Prime^®^ 2023.1.2. For assigning bacterial species, multilocus sequence typing (MLST) was performed using KmerFinder Database version 3.0.2 [13,29,30] and MLST 2.0 [31,32,33,34,35,36,37]. The identification of antimicrobial resistance genes and point mutations in CRE isolates was accomplished via the use of PointFinder and ResFinder version 4.1 [11,12,13]. Core genome alignments were generated via the alignment of short-read sequences to reference genome, *E. coli* str. K-12 substr. MG1655 (GenBank Accession: U00096) and *K. pneumoniae* CP000647. OmpC, OmpF, OmpK35, and OmpK36 amino acid changes were visualized and mapped to tertiary protein databank structures (7JZ3, 4GCS, 5o77, and 6RD3) using the molecular graphics program VMD [38]. *ompC* coverage gaps for all 29 *E. coli* Illumina paired-end-read files were trimmed, merged, normalized, and de novo assembled into contigs. Assembled contig lists of all 29 *E. coli* resulted in an average N50 of 59,489 base pairs (bp) long and an average sum contig length of 7,355,703 bp. Other assembly features are summarized in Supplement Table S4. Contig lists were dissolved and mapped against MG1655 K12 *E. coli* reference genome (accession: U00096).

### 4.3. Immunodetection and Sample Preparation

Samples from overnight growth in CAMH broth with ertapenem (1 ug/mL) were pelleted and solubilized in 200 uL water. Cell lysis was then accomplished via three rounds of freeze–thaw cycles, sonication, and boiling at 100 °C for 8 min. Bacterial protein lysate concentrations were determined with BCA Protein Assay Kit (Pierce), and samples were normalized to 1.2 ug/mL and then separated by electrophoresis with BIO-RAD Mini-PROTEAN TGX Stain-Free Gels with 4–15% polyacrylamide for an hour at 150V. Bands were transferred onto nitrocellulose membranes at 25 V for 50 min. Membranes were then blocked with 1% gelatin in 1× transfer buffer solution with tween (TBST) and anti-OmpC or anti-OmpF antibodies (ThermoFisher) overnight at 4 °C on a shaker. The membrane was then washed three times with ddH2O and subjected to a secondary antibody reaction with the BioRad Immuno-Blot Assay Kit (Goat anti-rabbit IgG) by diluting goat anti-rabbit secondary antibodies in gelatin buffer solution and rocking at 20 °C for 60 min. Membranes were washed three times with TBST solution and developed alkaline phosphatase.

In an effort to understand osmolarity-related effects of porin band intensity on included isolates, representative CRE clinical strains were grown in three different broths, ranging in osmolarity, including high salt Luria–Bertani Miller (LB) Millers broth (highest osmolarity), cation-adjusted Mueller–Hinton (CAMH) (low-moderate osmolarity), and nutrient broth (low osmolarity). No differences were seen in OmpC or OmpF bands between media, including in the ATCC reference strains (Supplement Data S2; Figure S3). Thus, CAMH was used solely for currently reported experiments. Four ertapenem- and meropenem-susceptible (EsMs) but ceftriaxone-resistant *E. coli* clinical isolates collected from blood sources were used as OmpC controls (Figure 3). In addition, ATCC 2340, a meropenem-resistant, *bla*_KPC_-producing CLSI control strain, was used as the reference for porin protein bands.

### 4.4. qPCR of β-lactamase Genes

To determine gene copy numbers, SYBR Green qPCR was performed using primers and a microplate reader (BioRad). The copy number was calculated using the formula ∆Ct = 2^(CTcontrol−CTtarget)^ and the mean plate Cq value for rpsL as the control gene [10,39]. Primers used for qPCR included a *bla*_CTX-M-15_ specific primer: 5′-ATGGATGAAAGGCAATACCA-3′ with an estimated amplicon size of 175 nucleotides (this study). In addition, a Group-1 *bla*_CTX-M_ primer: 5′-ATGGTTAAAAAATCACTGCG-3′ and Group-9 *bla*_CTX-M_ primer: 5′-ATGGTGACAAAGAGAGTGCA-3′ were used to both capture any addition *bla*_CTX-M_ genes within the groups as well as *bla*_CTX-M-14_ within Group-9 [40]. A *bla*_KPC_ primer was also used to screen ErMs isolates: 5′-TGTCACTGTATCGCCGTCTA-3′ (this study). Other primers included *bla*_OXA-1_: 5′- ACGTGGATGCAATTTTCTGT-3′ (this study), *bla*_SHV_: 5′-GCCGCTTGAGCAAATTAAAC-3′ (this study), *bla*_TEM_: 5′-CTGTTTTTGCTCACCCAGAA-3′ (this study), *E. coli* rpsL: 5′-ACCACCGATGTAGGAAGTCA-3′ (this study), and *K. pneumoniae* rpsL: 5′-GACCTTCACCACCGATGTAG-3′ (this study). All performed equally well, with 100% agreement with WGS data.

### 4.5. Sample Preparation for LC-MS/MS Analysis

The bacterial strains were grown on tryptic soy agar (TSA) with 5% sheep blood for 24 h at 37 °C. A single bacterial isolate was transferred to cation-adjusted Mueller–Hinton broth (CA-MHB). The cultures were incubated and shaken for 18 h. At the end of incubation, an (OD_600_) MacFarland 0.5 standard concentration was prepared with each inoculum. A meropenem–vaborbactam E-TEST strip (MEV [64/8 ug/mL]) was placed into a volume of 0.8 mL CA-MHB and then shaken for 30 min at 37 °C. Standardized inoculum was then transferred (0.2 mL) to each meropenem-vaborbactam-concentrated broth and incubated while shaken. At hours 1, 4, and 18, a 0.2 mL sample volume collection was taken from the test samples and centrifuged at 12,000 RPM at 4 °C for 10 min. At the end of the centrifugation, 100 uL volume of the resulting supernatant was collected and transferred to 300 uL ice-cold methanol and 15 uL of internal standard propranolol (IS) to a concentration of 5 ug/mL. Each tube was lightly vortexed by hand for 0.2 min and then placed on ice to incubate for 10 min. The remaining volume of the supernatant for each bacterial sample was carefully removed, and the resulting bacterial cell pellets were resuspended in 100 uL of PBS pH 7.4, sonicated for 5 min, and then were processed as were the collected 100 uL supernatant samples noted above for protein precipitation and pellet sample analysis. After ice incubation, samples were mixed and then centrifuged at 12,000× *g* at 4 °C for 10 min. A 100 uL volume of the supernatant was then transferred to 200 uL HPLC-grade water and mixed briefly. The sample was then transferred (150 uL) to an LC-MS/MS sample injection vial for analysis. Ertapenem and imipenem proved to be too labile to accurately detect at concentrations less than 128 mcg/mL. Meropenem remained stable at lower concentrations (<10 ng/mL). The sample analysis of the abundance of meropenem, inhibitor vaborbactam, and the selected (IS) internal standard propranolol were measured using an LC-MS/MS system comprising of a ACQUITY UPLC liquid chromatogram system and a Xevo TQD, tandem triple-quadrupole mass spectrometer by Waters corporation. Additional instrumentation parameters and analysis can be found in the Supplementary Materials.

### 4.6. Statistical Analyses

Student’s *t* test or the nonparametric Wilcoxon Rank Sum test was used for continuous variables based on distribution. The chi-square or Fisher’s Exact test was used to compare categorical variables. A two-sided *p*-value of less than 0.05 was considered statistically significant. All analyses were completed with R (v4.1.2).

## Figures and Tables

**Table 1:** Antimicrobial susceptibilities of carbapenem-resistant *E. coli* and *K. pneumoniae.*

Name	Overall (*n* 99) (%Susceptible)	ErMs (*n* 29) (%Susceptible)
Amikacin	91	65
Aztreonam	9	12
Ceftazidime–avibactam	77	88
Ciprofloxacin	9	12
Colistin	95	96
Doripenem	53	88
Doxycycline	44	38
Ertapenem	4	0
Cefepime	16	23
Cefotaxime	9	12
Gentamicin	39	48
Imipenem	46	73
Imipenem–relebactam	98	96
Levofloxacin	16	0
Meropenem	44	100
Meropenem–vaborbactam	88	88
Minocycline	63	68
Polymyxin B	95	96
Piperacillin–tazobactam	19	35
Trimethoprim-sulfamethoxazole	23	15
Ceftazidime	14	19
Tigecycline	98	96
Ticarcillin–clavulanic acid	11	15
Tobramycin	30	23

Antimicrobial susceptibilities of carbapenem-resistant *E. coli* and *K. pneumoniae* with microdilution assays (ThermoFisher (Waltham, MA, USA) [GNX2F]; CLSI M100-ED33:2023 Performance Standards for Antimicrobial Susceptibility Testing, 33rd Edition).

**Table 2:** Distribution of β-lactamase genes by species, phenotype, and carbapenemase status.

		Species			Carbapenem Phenotype			Carbapenemase Status		
*n* (%)	Overall (*N* = 76)	*K. pneumoniae* (*n* = 47)	*E. coli* (*n* = 29)	*p*	ErMr (*n* = 47)	ErMs (*n* = 29)	*p*	CPE (*n* = 35)	NCPE (*n* = 41)	*p*
ErMs	29 (38)	8 (17)	21 (72)	<0.001				5 (14)	24 (59)	<0.001
NCPE	41 (54)	19 (40)	22 (76)	0.01	17 (36)	24 (83)	<0.001			
CPE	35 (46)	28 (60)	7 (24)	0.01	30 (64)	5 (17)	<0.001			
*bla*_MBL_^A^	5 (7)	3 (6)	2 (7)	1.00	4 (9)	1 (3)	0.70	5 (14)	0 (0)	0.04
*bla*_KPC_^B^	28 (37)	23 (49)	5 (17)	0.01	24 (51)	4 (14)	0.002	28 (80)	0 (0)	<0.001
*bla*_OXA-48_^C^	2 (3)	2 (4)	0 (0)	0.70	2 (4)	0 (0)	0.70	2 (6)	0 (0)	0.41
*bla*_OXA-1/-9_	33 (43)	22 (47)	11 (38)	0.60	22 (47)	11 (38)	0.60	21 (60)	12 (29)	0.01
*bla*_ESBL_^D^	52 (68)	32 (68)	20 (69)	1.00	28 (60)	24 (83)	0.06	21 (60)	31 (76)	0.23
*bla*_CTX-M_^E^	47 (62)	27 (57)	20 (69)	0.45	23 (49)	24 (83)	0.01	16 (46)	31 (76)	0.02
*bla*_CTX-M-15_	43 (57)	27 (57)	16 (55)	1.00	22 (47)	21 (72)	0.05	16 (46)	27 (66)	0.13
*bla*_SHV-12_	7 (9)	7 (15)	0 (0)	0.08	4 (9)	3 (10)	1.00	4 (11)	3 (7)	0.83
*bla*_penicillinase_^F^	60 (79)	42 (89)	18 (62)	0.01	40 (85)	20 (69)	0.17	30 (86)	30 (73)	0.29
*bla*_AmpC_^G^	12 (16)	5 (11)	7 (24)	0.21	7 (15)	5 (17)	1.00	3 (8)	9 (22)	0.20

Distribution of β-lactamase genes based on short-read sequences. ErMs: ertapenem-resistant meropenem-susceptible, NCPE: non-carbapenemase-producing Enterobacterales, CPE: carbapenemase-producing Enterobacterales, ESBL: extended-spectrum β-lactamase. ^A^ Metallo-β-lactamases (MBL) variants: *bla*_NDM-1_, *bla*_NDM-5_, *bla*_VIM-27_. ^B^ *bla*_KPC_ variants: *bla*_KPC-2_, *bla*_KPC-3_. ^C^ *bla*_OXA-48_-like variants: *bla*_OXA-232_. ^D^ ESBL variants: *bla*_CTX-M-15_, *bla*_CTX-M-14_, *bla*_CTX-M-27_, *bla*_SHV-12_, *bla*_SHV-105_, *bla*_OXY-2-7_, *bla*_OXY-2-8_. ^E^ Any *bla*_CTX-M_: *bla*_CTX-M-15_, *bla*_CTX-M-14_, *bla*_CTX-M-27;_^F^ *bla*_penicillinase_: various *bla*_TEM-1_-like and *bla*_SHV-1_-like variants. ^G^ AmpC variants: *bla*_CMY-2_, *bla*_CMY-6_, *bla*_CMY-42_, *bla*_CMY-59_, *bla*_CMY-133_, *bla*_DHA-9_, *bla*_FOX-5._

**Table 3:** Meropenem hydrolysis across distinct beta-lactamase profiles.

ID	β-Lactamase Profile ^A^		Meropenem Hydrolysis (ng/mL-h)	Vaborbactam Hydrolysis (ng/mL-h) ^B^
	Carbapenemase	Non-Carbapenemase β-Lactamase		
EC68	none	*bla*_CMY-133_, *bla*_TEM-1_	−0.5	−0.1
EC5	none	*bla*_CTX-M-15_, *bla*_OXA-1_	−0.8	No loss
EC201	none	*bla*_CTX-M-15_, *bla*_OXA-1_	−1.0	No loss
KP56	*bla*_KPC-2_	*bla*_OXA-9,_*bla*_TEM-1_, *bla*_SHV-182_	−1.0	No loss
EC74	*bla*_KPC-3_	none	−1.3	No loss
KP15	*bla*_KPC-2_	*bla*_CTX-M-15_, *bla*_OXA-9,_*bla*_TEM-1_, *bla*_SHV-100_	−2.0	No loss
EC23	*bla*_NDM-5_	*bla*_CTX-M-15_, *bla*_OXA-1_, *bla*_TEM-1_, *bla*_SHV-27_	−2.8	No loss
EC22	*bla*_NDM-5_	*bla*_CTX-M-15_, *bla*_OXA-1_, *bla*_TEM-1_, *bla*_SHV-27_	LLQ at t_1_	No loss
KP26	*bla*_NDM-1_	*bla*_CTX-M-15_, *bla*_CMY-6_, *bla*_OXA-1_, *bla*_TEM-1_, *bla*_SHV-155_	LLQ at t_1_	No loss

EC: *E. coli*; KP: *K. pneumoniae*; LLQ: lower limit of quantitation; None: none detected; t_1_: hour 1 since drug exposure; t_2_: hour 18 since drug exposure. ^A^ β-lactamase profile determined by short, raw read uploads to ResFinder database [11,12,13]. ^B^ Vaborbactam concentrations remained constant between hours 1 and 18 across all nine isolates with an average t_2_ − t_1_ concentration of +0.75 ng/mL and overall average parent concentration of 6.0 ng/mL at all time points.

**Table 4:** Mean ΔCt of resistance genes relative to *rpsL* among *E. coli* and *K. pneumoniae* ErMs and EsMs.

Phenotype	ID	*rpsL*	*bla*_CTX-M_	*bla_TEM_*	*bla_SHV_*	*bla_OXA-1/9_*	*bla_CMY_*	*bla*_KPC_	*ompC/* *ompK36*	*ompF/* *ompK35*	*tolC/* *oqxA*
ErMs	EC12	1.0	2.3	0.4		0.6		1.6	0.5	0.4	0.4
	EC30		48.8	2.8					1.4	1.3	1.7
	EC31		5.2	0.9					0.6	0.6	0.8
	EC35		39.9	1.2			0.1		0.7	0.9	1.0
	KP10	1.0	10.7	4.7	0.5	4.3			0.6	0.7	0.7
	KP38		20.4	4.3	1.6				1.7	1.8	1.9
	KP45		9.1	4.9	9.2				1.2	1.3	1.0
	KP54			0.2	0.2	0.2		0.2	0.2	0.3	0.2
	Mean ΔCt		17.1	2.4	1.4	0.6	0.0	0.2	0.9	0.9	1.0
EsMs	EC87	1.0	1.6			0.7			0.6	0.5	0.6
	EC88		8.1						0.8	0.6	1.9
	EC89		2.1	2.0					0.6	0.7	1.5
	EC92		1.4			0.6			0.5	0.4	1.2
	KP85	1.0	1.3	1.2	0.8	0.5			0.5	0.5	
	KP86		2.7		0.7		0.3			0.4	0.6
	KP90		20	8.6	1.0	3.6			0.8	1.0	1.6
	KP91		1.1				0.1			0.4	0.4
	Mean ΔCt		4.8	1.5	0.3	0.7	0.1	0.0	0.5	0.6	1.1

Mean fold gene copy number relative to *rpsL* (species-specific) of ertapenem-resistant but meropenem-susceptible (ErMs) and ceftriaxone-resistant but ertapenem- and meropenem-susceptible (EsMs) *E. coli* and *K. pneumoniae*. Fold copies calculated with formula ΔCt = 2^(CTrpsL−CTtarget)^ relative to each isolate. Group-1 and Group-9 *bla*_CTX-M_ primers used for screening. Porin and efflux genes, *ompC*/*ompF*/*tolC* and *ompK35*/*ompK36*/*oqxA*, were analyzed in *E. coli* and *K. pneumoniae*, respectively. KP54 is ATCC ErMs strain BAA-1903 with a subpopulation of KPC producers.

**Table 5:** Major amino acid alterations in porin genes in *E. coli* and *K. pneumoniae* by carbapenemase status and carbapenem phenotype.

	*E. coli*			*K. pneumoniae*		
	CP-ErMr (*n* = 2)	NCP-ErMs (*n* = 16)	*p*	CP-ErMr (*n* = 28)	NCP-ErMs (*n* = 8)	*p*
No major alteration(s)	2 (100)	0 (0)	0.002	1 (3.6)	0 (0)	1.00
Any major alteration(s)	0 (0)	16 (100)	0.002	27 (96)	8 (100)	1.00
*ompC*/*ompK35*	0 (0)	14 (88)	0.05	27 (96)	8 (100)	1.00
*ompF/ompK36*	0 (0)	8 (50)	0.55	20 (71)	4 (50)	0.47
Insertion/Deletion	0 (0)	10 (63)	0.85	27 (96)	8 (100)	1.00
*ompC*/*ompK35*	0 (0)	10 (63)	0.85	27 (96)	8 (100)	1.00
*ompF/ompK36*	0 (0)	0 (0)	ND	0 (0)	0 (0)	ND
Frameshift	0 (0)	16 (100)	0.002	27 (96)	8 (100)	1.00
*ompC*/*ompK35*	0 (0)	14 (88)	0.05	24 (85)	8 (100)	0.62
*ompF/ompK36*	0 (0)	8 (50)	0.55	0 (0)	0 (0)	ND
Premature Stop	0 (0)	1 (6.2)	1.00	25 (89)	8 (100)	0.80
*ompC*/*ompK35*	0 (0)	1 (6.2)	1.00	23 (82)	7 (87)	1.00
*ompF/ompK36*	0 (0)	0 (0)	ND	20 (71)	4 (50)	0.47

Major amino acid alterations in porin genes in *E. coli* and *K. pneumoniae* by carbapenemase status and carbapenem phenotype determined by short-read sequences mapped to reference. Abbreviations: CP: carbapenemase-producing; ErMs: ertapenem-resistant, meropenem-susceptible; ErMr: ertapenem- and meropenem-resistant; NCP: non-carbapenemase-producing; ND: not detected. Major alterations in either *ompF*-like or *ompC*-like genes are included unless specific gene noted.

## Data Availability

Data supporting results have been deposited in the NCBI BioProject (PRJNA1049776).

## Supplementary Materials

The following supporting information can be downloaded at https://www.mdpi.com/article/10.3390/antibiotics13020185/s1. Supplemental Data S1: annotated mobile genetic elements and counts of included ErMs and EsMs; Supplemental Data S2: ErMs vs. ErMr mobile genetic element count volcano plot; OmpC alteration sites on modeled structure; SDS-PAGE and immunodetection results; LC-MS/MS analysis concentrations at time points; Standard curve and quality control/sample preparation for LC-MS/MS analysis; in vivo sources of collected ErMs *E. coli* and *K. pneumoniae* isolates; Distribution of porin amino acid alterations among *E. coli* and *Klebsiella* spp.; Mapped reads of ErMs *E. coli* (EC30) with coverage gap in *ompC*; Correlation of *ompC* and *bla*_CTX-M_ ∆Ct relative to *rpsL* across EsMs and ErMs; antimicrobial susceptibility and EsMs *E. coli*. Supplemental Data S3: LC-MS/MS standard curve and parent molecule concentration calculations; Supplemental Data S4: Contig details of assembled ErMs *E. coli* used for *ompC* mapping.
