# Supplementary material for: Diverse Role of blaCTX-M and Porins in Mediating Ertapenem Resistance among Carbapenem-Resistant Enterobacterales
Source: Antibiotics (Basel). 2024 Feb 13;13(2):185. doi: 10.3390/antibiotics13020185 (PMC10885879; doi:10.3390/antibiotics13020185)
Supplement: Supplementary file 1 [file antibiotics-13-00185-s001.zip › Supplemental Data S2.zip!/Supplemental Data S2.pdf]

Supplementary Data and Figures

Diverse Role of *bla*$_{CTX-M}$ and Porins in Mediating Ertapenem Resistance among Carbapenem Resistant Enterobacterales

Cody A. Black, Raymond Benavides, Sarah M. Bandy, Steven D. Dallas, Gerard Gawrys, Wonhee So, Alvaro G. Moreira, Samantha Aguilar, Kevin Quidilla, Dan F. Smelter, Kelly R. Reveles, Christopher R. Frei, Jim M. Koeller and Grace C. Lee

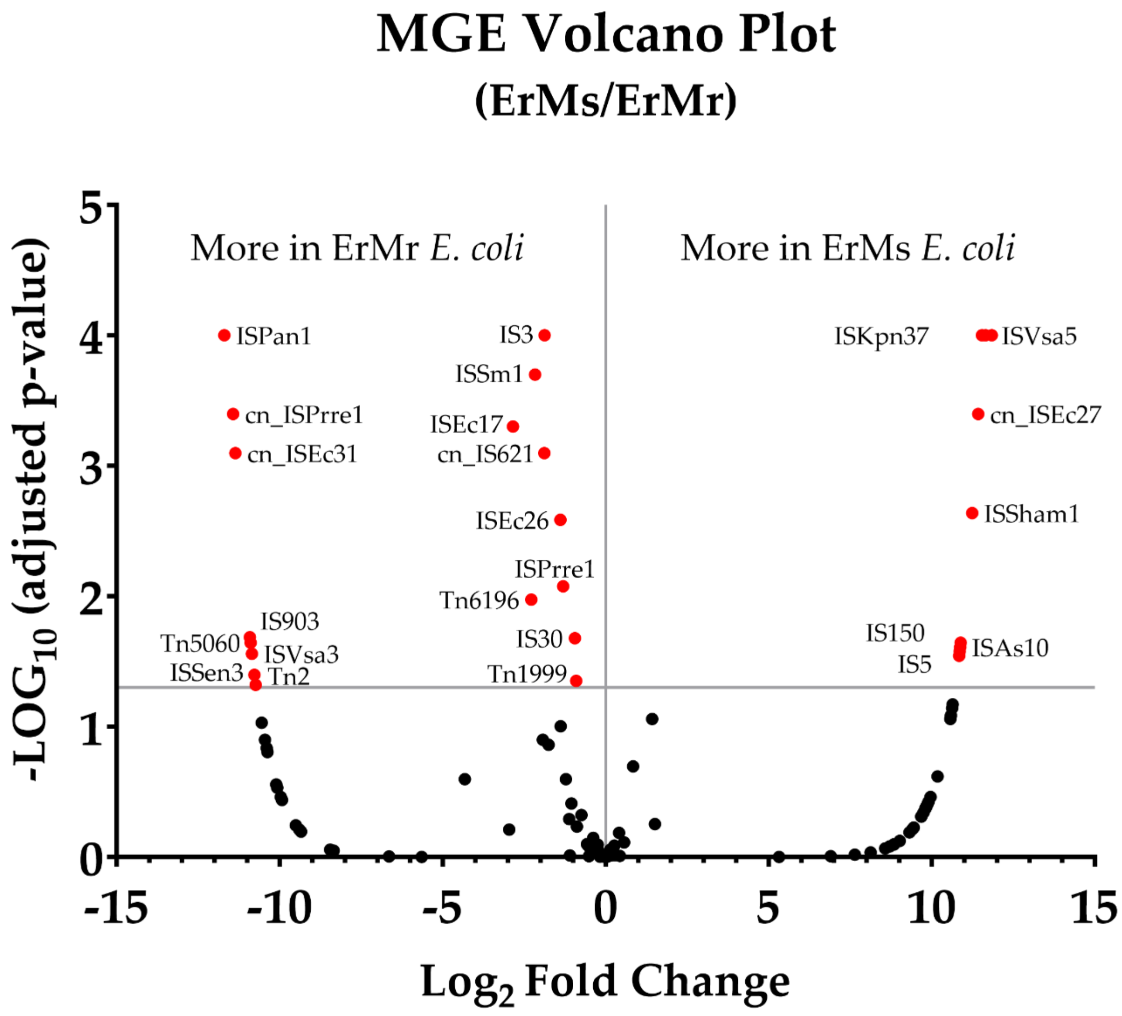

Figure S1. Volcano plot comparing all MGE counts between EsMs and ErMs. Log2-fold count difference between ErMs and EsMs MGEs were plotted against Log10-transformed adjusted *p-values* (two-way ANOVA) of all MGEs between these two phenotypes. Values above 1.3 Log10 (p < 0.05; grey line) were considered statistically significant. All red MGEs are present at higher frequencies in ErMs than EsMs *E. coli*.

OmpC alteration sites on modeled structure

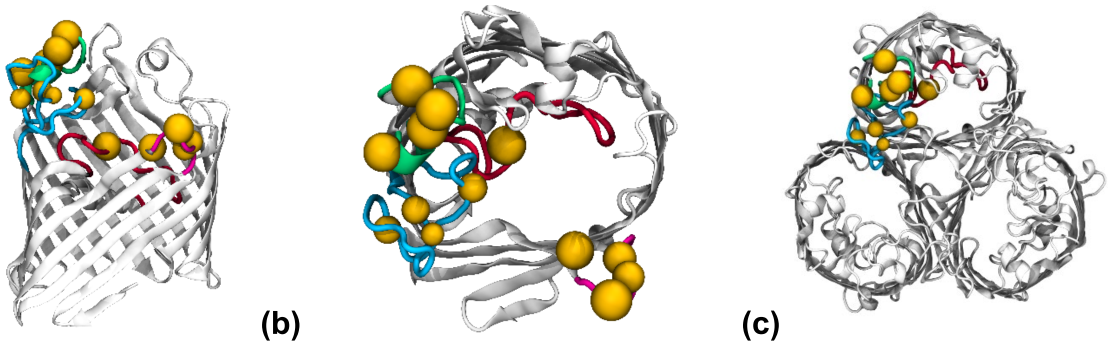

(a) (b) (c)

Figure S2. OmpC porin visualization with most frequent sites of amino acid sequence alterations among non-carbapenemase producing *E. coli* and *K. pneumoniae* (OmpK36 homologue). (a) Side view of OmpC monomer, extracellular side facing up with key regions highlighted. (b) Top view from extracellular side. (c) Top view of trimer formation with key regions highlighted in only one monomer. Colors: loop 2 (pink), loop 3 (red), loop 4 (blue), loop 5 (green) and sites of alterations shown as gold spheres. The size of the spheres relates to the frequency of occurrence (bigger sphere = more frequent). Visualization and rendering performed with VMD [9]. PDB: 7JZ3, wildtype OmpC from *E.coli* K12.

Loops 1, 2, 4, 5, 6, 7, and 8 all face the extracellular space, however, as native porins exist as trimeric units, the specific roles of each loop ranges from anchoring neighbor monomers to ligand binding or epitope formation. However, Loop 3 has a unique function, as it folds into the channel of the pore, forming a charged constriction zone which is lined by acidic residues from the loop and basic residues from the adjacent β-strands 1, 2, 4, and 6. Outside of major mutations which prevent or drastically decrease the translation of functional porins (e.g., premature stop codons, insertion sequence disruption), Enterobacterales seem to tolerate various amino acid changes within their porin sequences which may result in ertapenem non-susceptibility, especially in conjunction with β-lactamase overproduction, depending on the specific species.

SDS-PAGE and immunodetection

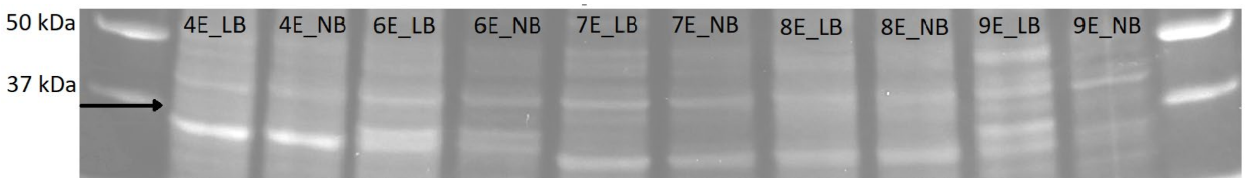

SDS-PAGE relative band intensities:

isolate_broth	lane_signal	ompa_signal	ompa:lane	ompa_norm	ompf_signal	ompf:lane	ompf_norm	ompc_signal	ompc:lane	ompc_norm
4E_LB	32913157	1223416	0.037171032	1	1116852	0.033933299	1	1124336	0.034160685	1
4E_NB	32752353	1184931	0.0361785	0.973298246	1072722	0.032752517	0.965202852	1071336	0.032710199	0.957539322
6E_LB	33731156	1123576	0.033309739	0.896120916	1097395	0.032533572	0.958750641	1068344	0.031672321	0.927157071
6E_NB	30225027	899684	0.029766193	0.800790081	669000	0.022133975	0.652278908	688971	0.022794719	0.667279326
7E_LB	34392924	1160031	0.033728769	0.907393953	679866	0.019767613	0.582543201	787540	0.022898315	0.670311926
7E_NB	31455415	1024830	0.0325804	0.876499749	616317	0.019593351	0.577407796	790813	0.025140759	0.735955924
8E_LB	32507954	873717	0.026877022	0.723063659	827631	0.025459338	0.750275954	859086	0.026426948	0.77360707
8E_NB	32357415	996764	0.030804809	0.828731622	933942	0.028863307	0.850589471	967841	0.029910949	0.875595703
9E_LB	31846292	812247	0.02550523	0.686158786	828376	0.026011694	0.76655364	812375	0.025509249	0.7467429
9E_NB	30548216	790624	0.025881184	0.696272955	764518	0.025026601	0.737523355	813710	0.026636907	0.779753307

Figure S3. SDS-PAGE revealed similar protein profiles of the included isolates with some differences shown in figure below. Specifically, 7E (LB/NB), 8E (LB/NB), and 9E (NB only) seem to have a loss of a band around 37 kDa when compared to the rest of the lanes. The effect of osmolarity difference between the two growth medias did not seem to cause dramatic differences between within the same isolate, except isolate 9E which seemed susceptible to porin loss at low osmolarity (NB). Visually determined turbidity scores between 0 (no growth) to 4 (full growth) were given to each overnight growth at 18 hours of incubation. All LB-grown isolates scored between 3-4 while NB-grown isolates received a score of 2-3. ATCC 25922 was used as control (4E).

Standard Curve and QC. sample preparation for LC-MS/MS analysis.

Preparation of samples for a standard curve and controls for detection of carbapenemase activity using mass spectrometry were similar in development of the test samples. A prelabeled 15mL conical bottom tissue culture tube and an antimicrobial test strip (MEV, [64/8ug/mL]), (Meropenem plus Vaborbactam) combo test strip was placed into a tube. Where a volume of 1mL of the cation adjusted Muller Hinton growth media was added to the tube and recap well. The media solvent was allowed to solubilize off the drug while shaking horizontally for 30 minutes. With the use of serial dilution step, a 0.5mL volume of it was carried over to a new 15mL tube containing 0.5mL Muller Hinton and was mix by vortex for 0.2 minutes. This was continued four more times with a series of tubes to complete a series of Standard Curve samples concentrations of [S5 ~ S1], MEV (Meropenem and Vaborbactam); [64/8ug/mL], [32/4 ug/mL], [16/2 ug/mL], [8/1 ug/mL] and [4/0.5 ug/mL]. For the use of QC samples, a separate preparation of dilutions was made and the selection of the [32/4 ug/mL] and [8/1 ug/mL] were selected and used as QC High and QC Low.

The sample extraction of standard curve and QC samples for LC-MSMS analysis were prepared as followed in the test samples preparation as noted above.

A 0.2mL standard curve sample were taken from the assigned standard curve test sample and transferred to pre labeled 1.8mL micro vial. To remove any solid material before protein precipitation, each tube was centrifuge at 12,000rpm at 4^0C for 10 minutes. At the end of the centrifugation, 100uL volume of the resulting supernatant was collected and transfer to a new pre labeled 1.8mL micro vial containing 300uL ice cold methanol and 15uL (IS) internal standard Propranolol [5ug/mL] concentration. Each tube was lightly vortex by hand for 0.2 minutes then placed on ice to incubate for 10 minutes.

After ice incubation, each were mix by vortex by hand for 0.2 minutes then transferred to a centrifuge and centrifuge all tubes at 12,000g at 4^0C for 10 minutes. At the end of centrifugation, a 100uL volume of the supernatant was transfer to a pre labeled 1.5mL micro vial containing 200uL HPLC grade H2O and were mix by vortex for 0.1 minutes. A 150uL volume of the sample was then transferred to a pre labeled LC-MSMS sample injection vial and a 10uL volume was used for LC-MSMS analysis.

Instrumentation and LC-MS/MS parameters and Analysis

The sample analysis of the abundance of Meropenem, inhibitor Vaborbactam and the selected (IS) internal standard Propranolol were measured using a LC-MS/MS system comprising of a ACQUITY UPLC liquid chromatogram system and a Xevo TQD, tandem triple-quadrupole mass spectrometer by Waters corporation.

Chromatography separation was preformed using an ACUITY UPLC (2.1x100mm) (CSH) Phenyl-Hex (1.7um) column make up manufacture by WATERS corporation column. The flow rate was 0.3mL/minute and the column temperature were set to 40^0C. The sample injection volume for the LC-MS/MS was set to 10uL. The mobile phase consisted of 0.1% Formic acid in water (A) and 0.1% Formic acid in (ACN) acetonitrile (B). A gradient set up to run, with the initial gradient concentration started at (A) 70% and (B) 30% and set to reverse the concentrations the end of 5 minutes. An initial column equilibration was set to run at (A) 70% and (B) 30% for 2 minutes before the next injection.

Ionization was achieved using electrospray in the positive ionization mode (ESI^+) for Meropenem and Propanol internal stand but (ESI-) negative mode ran best for Vaborbactam with their electrospray voltages setting. Nitrogen was used as the sheath and auxiliary gas at 28 units and 2 units (arbitrary units). The heated capillary temperature was 400^0C and the argon collision gas pressure was set to 1 mTorr.

The multiple-reaction-monitored (MRM) parameters were optimized by post column infusion for each compound stock solution of [1ug/mL] using the manual and IntelliStart auto-tune software.

The optimal MRM parameters are summarized in the section titled “Mass Spectrometer LC-MS/MS Conditions Table 1” listed below.

UPLC Conditions:

UPLC (Mobile Phase and Analytical Column Used for Sample analysis)

(Mobile Phase)
A = (Aqueous system) H2O with (0.1% Formic Acid)
B = (Organic system) (ACN) Acetonitrile with (0.1% Formic Acid)
UPLC System Flow Rate: set to [0.3 mL/min.]

(Analytical Column Used: Analytical Waters Corp.)
ACQUITY UPLC (2.1x100mm) (CSH) Phenyl-Hex (1.7um) column by WATERS
(Part No. 186005407); (Lot No. 0124311401)
(Guard Column:) (Column In-Line Filter Unit, Waters ACQUITY UPLC System)
(Filter, 2.1mm), (2.1um), (Waters No. P/N 289002078)

(InLet Method: Settings for assay run)
(UPLC/ InLet Method / Waters Acquity SDS Method)
The following timetable describes the concentration of the mobile phase gradients, the flow of the stream to the MS and the contact closure switch to the UPLC system.

#	Time (min.)	Flow Rate (mL/min.)	% Solvent A	% Solvent B	Gradient Curve 6
1	Initial	0.30	70.0	30.0	6
2	1.00	0.30	70.0	30.0	6
3	2.00	0.30	70.0	30.0	6
4	2.50	0.30	30.0	70.0	6
5	3.50	0.30	30.0	70.0	6
6	4.00	0.30	70.0	30.0	6
7	5.00	0.30	70.0	30.0	6

Injection Volume: (10uL)
Total Run Time: (5.0 minutes)

Flow Rate: (0.30 mL/min.)
Analytical Column Temperature: (40^0C)

Mass Spectrometer (LC/MSMS) Conditions (Table#1):

(MS Tune File: Method Settings for Each Assay Run)
Waters Xevo TQD: Tune Parameters
(Accusation Control Software used: **MassLynx (V4.1 SCN 882)** Control Settings:

Source (ES+)	{Setting}
Capillary (kV)	4.00
Cone (V)	38.00
RF (V)	2.50
Extractor (V)	3.00
Source Temp. (°C)	150
Desolvation Temp. (°C)	500
Cone Gas Flow (L/Hr.)	150

Source (ES+)	{Setting}
Desolvation Gas Flow (L/Hr.)	650
Collision Gas Flow (mL/Min)	ON
MS Mode Collision Energy	3.00
MSMS Mode Collision Energy	20.00
Pressure Gauge Collision Cell Pressure (mbar)	3.64e-003

(MS File: Method Settings for Each Assay Run)

Compound	Function #	Mode Type	Precursor Ion	Ion Mode
**Meropenem**	**1**	MRM	$[M+H]^+$	ES+
**Vaborbactam**	**2**	MRM	$[M-H]^-$	ES-
**(IS) Propanol**	**3**	MRM	$[M+H]^+$	ES+

MRM of {Parent to Daughter Ion}					
**Compound**	**(Prt.)**	**(Dau.)**	**Dwell**	**Cone (V)**	**Coll. (eV)**
**Meropenem**	**384.09**	*(67.87)* **(140.90)** *(113.81)*	**0.025**	**38.00**	**20.00**
**Vaborbactam**	**295.97**	*(67.88)* **(233.90)** *(277.90)*	**0.025**	**40.00**	**20.00**
**(IS) Propanol**	**260.18**	**116.00**	**0.025**	**35.00**	**20.00**

(Monitor Mass-to-Charge ratio (m/z) transition ions)

Meropenem (m/z transition of 384.09 > 140.90)
Vaborbactam (m/z transition of 295.97 > 233.90)
(IS) Propanol (m/z transition of 260.18 > 116.00)

Calculations:

The data is to be calculated using the WATERS TargetLynx Method Editor program. The established program is set to specify the parameters that control and measure the integration, calibration, and quantitation of the resulting peak areas in the Standard Curve, QC, and study samples. The standard curve is weighted 1/x2. The standard curve must have an r2 of 0.99 or greater. Study sample duplicate values must be within 15% of each other. Values outside these ranges must be repeated.

QCs, in a bacterial extracted sample run, sample QC values must not exceed the theoretical values by no more than: QC (High) [32/4 ug/mL] =<15% QC (Low) [8/1 ug/mL] =< 15%

If both values of any one QC sample fall outside of these ranges; the run must be repeated.

Table S1. LC-MS/MS analysis Concentrations at Time Points

ID	Species	MLST	β-Lactamase Gene Carriage[A]	Porin Gene Alteration(s)[B]	Time (hours)	Meropenem (ng/mL)
6E	*E. coli* (CPE/ ErMs-like)	131	*bla*$_{KPC-3}$	*ompc*-WT *ompf*-WT	1	33.7
					18	11.35
7E	*E. coli* (NCPE)	2	*bla*$_{TEM-1}$, *bla*$_{CMY-133}$	*ompc*-WT *ompf*-WT	1	21.1
					18	13.17
8E	*E. coli* (NCPE)	2	*bla*$_{OXA-1}$, *bla*$_{CTX-M-15}$	*ompc*-fs *ompf*-ps	1	29.4
					18	15.97
9E	*E. coli* (CPE)	2	*bla*$_{NDM-5}$, *bla*$_{OXA-1}$, *bla*$_{CTX-M-15}$, *bla*$_{TEM-1}$, *bla*$_{SHV-27}$	*ompc*-ms *ompf*-WT	1	0
					18	0
10E	*E. coli* (CPE)	2	*bla*$_{NDM-5}$, *bla*$_{OXA-1}$, *bla*$_{CTX-M-15}$, *bla*$_{TEM-1}$, *bla*$_{SHV-27}$	*ompc*-ms *ompf*-delins	1	60.7
					18	13.05
11K	*K. pneumoniae* (CPE)	307	*bla*$_{KPC-2}$, *bla*$_{OXA-9}$, *bla*$_{CTX-M-15}$, *bla*$_{TEM-1}$, *bla*$_{SHV-100}$	*ompk35*-fs/ps *omp36*-delins/Loop 3 GGdelins	1	39.1
					18	5.17
12K	*K. pneumoniae* (CPE, ATCC 1705)	258	*bla*$_{KPC-2}$, *bla*$_{OXA-9}$, *bla*$_{TEM-1}$, *bla*$_{SHV-182}$	*ompk35*-fs/ps *ompk36*-deins/fs	1	21.9
					18	4.97
13K	*K. pneumoniae* (CPE)	11	*bla*$_{NDM-1}$, *bla*$_{OXA-1}$, *bla*$_{CTX-M-15}$, *bla*$_{TEM-1}$, *bla*$_{SHV-155}$, *bla*$_{CMY-6}$	*ompk35*-ps *ompk36*-delins/fs/ps	1	0
					18	0
14E	*E. coli* (NCPE/ ErMs-like)	ND	*bla*$_{CTX-M-15}$, *bla*$_{OXA-1}$	ND	1	29.8
					18	12.45

Table S2. LC-MS/MS analysis Concentrations of Suspensions of Meropenem and Vaborbactam at Time Points

ID	LC-MS/MS Meropenem (ng/mL) in Suspension			LC-MS/MS Vaborbactam (ng/mL) in Suspension		
	1 hour	2 hour	18 hour	1 hour	2 hour	8 hour
**EC74**	33.7	32.6	11.35	4.8	4.3	5.53
**EC5**	29.4	36.3	15.97	6.7	5.4	7.64
**EC201**	29.8	48.1	12.45	6.2	6.3	7.87
**EC68**	21.1	31.4	13.17	6.8	5.1	5.36
**EC22**	llq	llq	llq	6.1	5.1	7.83
**EC23**	60.7	42	13.05	6.1	6.4	7.04
**KP26**	llq	llq	llq	4.6	4.8	6.18
**KP15**	39.1	35.9	5.17	7	6.1	8.11
**KP56**	21.9	21.8	4.97	3.6	3	3.14

Table S3. In-vivo sources of collected ErMs *E. coli* and *K. pneumoniae* isolates

**Source**	**# of ErMs**
urine	33
tissue	8
Body fluid	8
blood	7
sputum	6
unknown	12

Distribution of Porin Amino Acid Alterations among *E. coli* and *Klebsiella* spp.

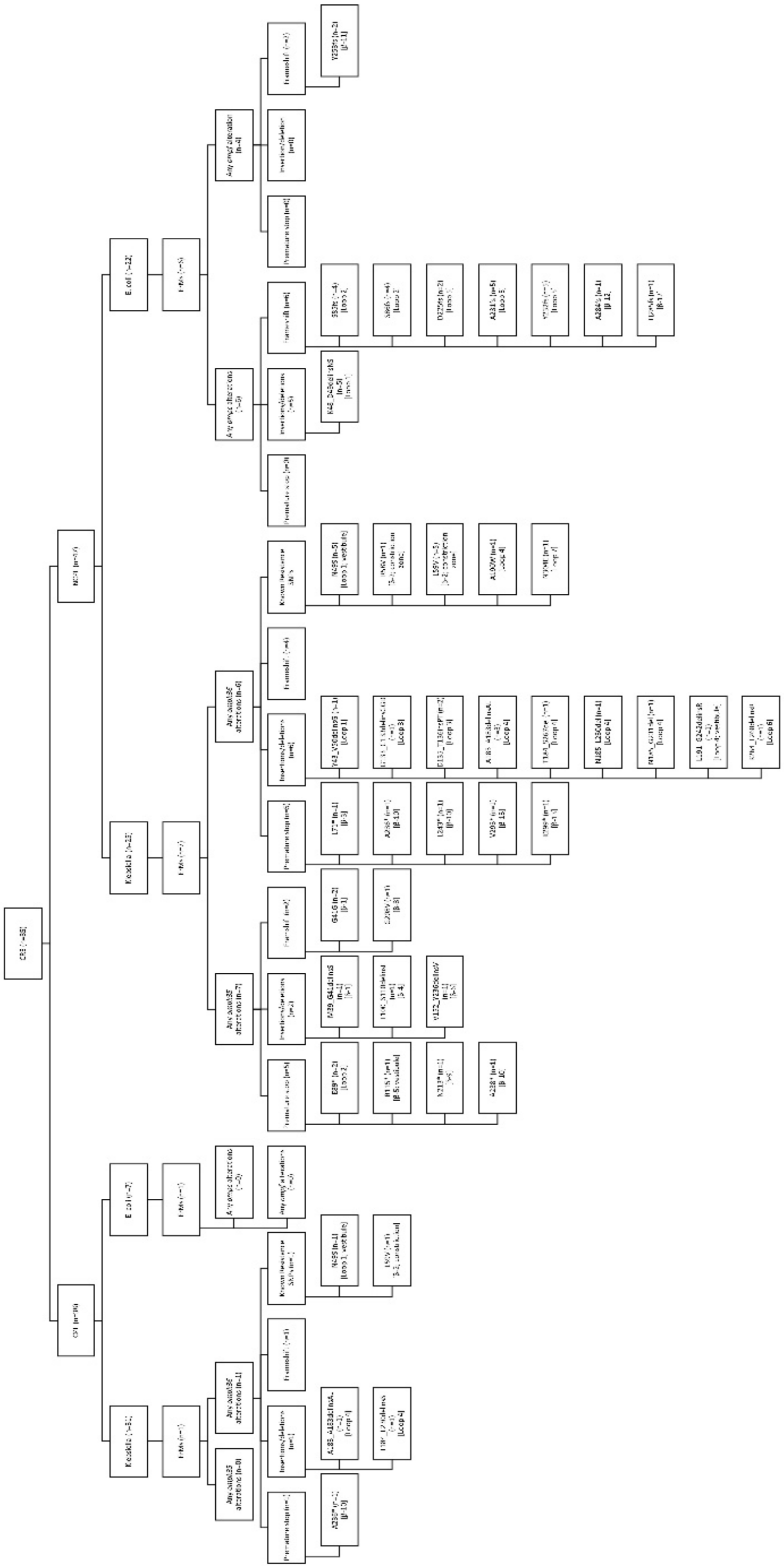

Mapped reads of ErMs *E. coli* (EC30_1022014) with coverage gap in *ompC*.

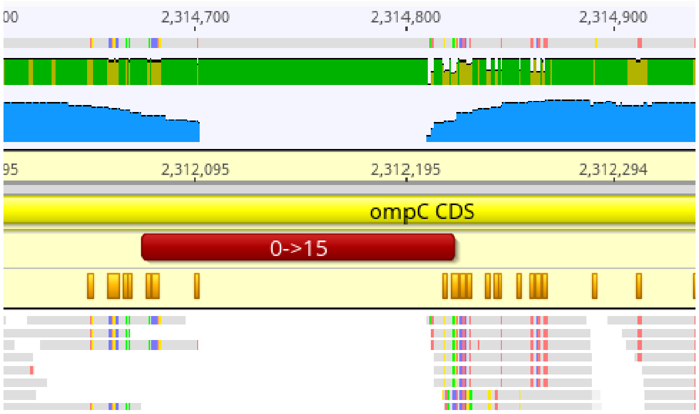

Figure S4. Coverage gap defined as areas where coverage falls below two standard deviations from the mean coverage. Coverage (blue): The number of non end-gap characters at each position. The scale bar has an indicator at the mean coverage level. Mean pairwise identity over all pairs in the column: Green: 100% identity, Green-brown: at least 30% and under 100% identity, Red: below 30% identity. Yellow: reference *ompC* gene; Orange: variant track.

Figure S5. Correlation of *ompC* and *bla*$_{CTX-M}$ ΔCt relative to *rpsL* across EsMs and ErMs

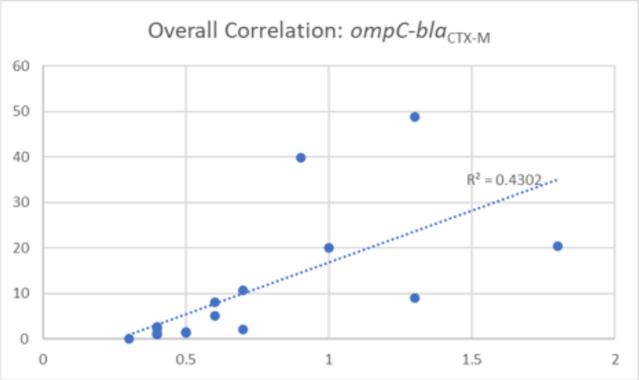

Phenotype	ID	*rpsL*	*ompC*/ *ompK36*	*bla*$_{CTX-M}$
ErMs	EC12	1	0.5	2.3
	EC30		1.4	48.8
	EC31		0.6	5.2
	EC35		0.7	39.9
	KP10	1	0.6	10.7
	KP38		1.7	20.4
	KP45		1.2	9.1
	KP54		0.2	0
EsMs	EC87	1	0.6	1.6
	EC88		0.8	8.1
	EC89		0.6	2.1
	EC92		0.5	1.4
	KP85	1	0.5	1.3
	KP86		0	2.7
	KP90		0.8	20
	KP91		0	1.1

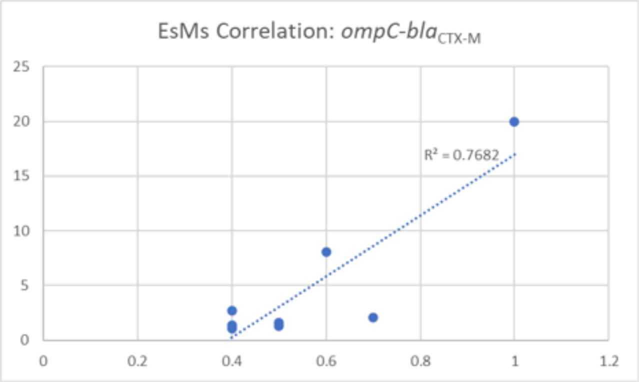

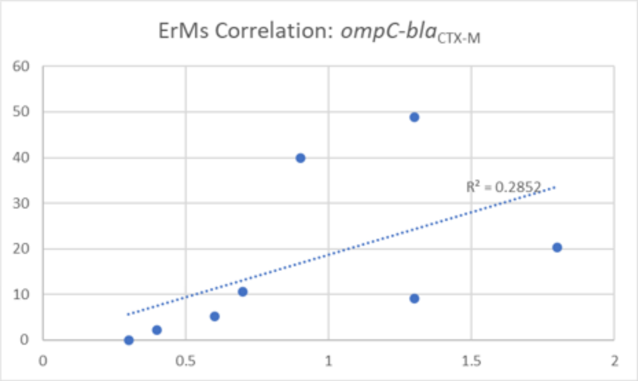

Table S4. Antimicrobial susceptibility and EsMs *E. coli*

Isolate	Ceftriaxone MIC (mcg/mL)	Ertapenem MIC (mcg/mL)	Meropenem MIC (mcg/mL)
EC87 (171)	>=64	<=0.5	<=0.25
EC88 (172)	>=64	<=0.5	<=0.25
EC89 (173)	>=64	<=0.5	<=0.25
EC92 (177)	>=64	<=0.5	<=0.25
KP85 (169)	>=64	<=0.5	<=0.25
KP86 (170)	>=64	<=0.5	<=0.25
KP90 (174)	>=64	<=0.5	<=0.25
KP91 (176)	>=64	<=0.5	<=0.25
